# Self‐Powered Smart Textile Based on Dynamic Schottky Diode for Human‐Machine Interactions

**DOI:** 10.1002/advs.202207298

**Published:** 2023-02-13

**Authors:** Pengfei Deng, Yanbin Wang, Ruizhe Yang, Zijian He, Yuanqiu Tan, Zhihong Chen, Jun Liu, Tian Li

**Affiliations:** ^1^ School of Mechanical Engineering Purdue University West Lafayette IN 47907 USA; ^2^ Department of Mechanical and Aerospace Engineering University at Buffalo The State University of New York Buffalo NY 14260 USA; ^3^ RENEW (Research and Education in Energy Environment and Water) Institute University at Buffalo The State University of New York Buffalo NY 14260 USA; ^4^ Elmore Family School of Electrical and Computer Engineering Purdue University West Lafayette IN 47907 USA

**Keywords:** biaxial detection, dynamic Schottky diodes, self‐powered, sensing network, smart textile

## Abstract

The growing demand for sustained self‐powered devices with multifunctional sensing networks is one of the main challenges for smart textiles, which are the critical elements for the future Internet of Things (IoT) and Point of Care (POC). Here, cellulose‐based smart textile is integrated with dynamic Schottky diode (DSD) to generate sustained power source (current density of 8.9 mA m⁻^2^) for self‐powered built‐in sensing network. In response to normal and shear motions, a pressure sensor with a sensitivity of 0.12 KPa⁻^1^ and an impact sensor are demonstrated, respectively. The woven structure of the textile contributes to signal amplification, which can also form a matrix of sensing elements for distributed sensing. The proposed strategy of fabricating self‐powered and multifunctional sensing networks with smart textiles shows tremendous potential for future intelligent society.

## Graphical abstract

A self‐powered sensing network is built into primary textiles, providing an ideal platform for spatial pressure and shear motion distribution recognition. The device opens new opportunities for the development of wearable electronics in next‐generation smart textiles and future intelligent society, enabling techniques to integrate sensor systems comfortably into primary clothing, shoes, carpet, etc.

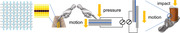

## 1 Introduction

Wearable electronics have attracted increasing attention due to their flexibility, portability, and functionalities in potential future applications like the Internet of Things (IoT)^[^ ^1^, ^2^, ^3^ ^]^ and Point of Care (POC).^[^ ^4^, ^5^, ^6^ ^]^ Despite intensive efforts in the field, the growing demand for sustained self‐powered and built‐in sensing networks that feature functionalities responding to user input and environmental stimuli remains one of the main challenges. In traditional wearable systems, sensing units and power units are usually designed separately, where additional power sources are needed, leading to extra and burdensome occupations and difficulties in coordinating the merits of devices with excellent wearability. Even though flexible materials are developed for wearable devices, they usually serve as a substrate or modified substrate, and additional rigid functional components are still needed. Therefore, revolutionary new materials and techniques are required for the development of the integrated sensing system with decent mechanical deformability and durability.

Thanks to their comfort, lightweight, and washability, smart textiles have emerged as competitive candidates for next‐generation wearable devices, including multifunctional sensors,^[^ ^7^, ^8^ ^]^ e‐skin,^[^ ^9^, ^10^ ^]^ health monitoring,^[^ ^11^, ^12^ ^]^ energy generation^[^ ^13^, ^14^ ^]^ and display systems.^[^ ^15^, ^16^ ^]^ Meanwhile, harnessing multiple functionalities with the fiber materials, such as electronic,^[^ ^17^ ^]^ optoelectronic,^[^ ^18^ ^]^ and acoustic,^[^ ^19^ ^]^ represents an intriguing way to achieve innovative and intelligent textiles. Among all raw materials for fibers and textiles, cellulose shows promising potential in the development of wearable devices owing to its biocompatible, biodegradable, environmentally‐friendly, sustainable, and stretchability. Mechanical energy harvesting technologies, such as triboelectric nanogenerators (TENG),^[^ ^20^, ^21^ ^]^ piezoelectric nanogenerators,^[^ ^22^, ^23^ ^]^ and electrochemical–mechanical generators,^[^ ^24^, ^25^ ^]^ that can directly convert mechanical energy into electrical power become promising solutions for self‐powered integrated smart textile. In recent years, semiconductor‐based direct‐current (DC) nanogenerators have been proposed with exceptional performance due to their high current density, simple structure, and excellent efficiency at all‐frequency spectrum without additional bulky and ridged rectification,^[^ ^26^, ^27^, ^28^ ^]^ which distinguish themselves from other alternating current (AC) nanogenerators. Yang et al.^[^ ^29^ ^]^ demonstrated a flexible tribo‐tunneling DC power generation based on a metal/conducting polymer sliding system with high output performance (> 200 µA). Meng et al.^[^ ^30^, ^31^ ^]^ reported flexible textile‐based DC generators with dynamic metal‐semiconducting polymer contact, offering an efficient strategy for harvesting mechanical energies.

Nevertheless, the lack of realization of sensing networks in response to external input in multiple directions and types hinders its further applications in the future of IoT and POC. These limitations remain in leveraging and incorporating DC generator performance for long‐term built‐in multifunctional sensing networks in conjunction with smart textiles with great wearability and durability. Therefore, the development of the built‐in sensing network in cellulose‐based smart textiles and the investigation of mechanisms and solutions for long‐lasting Dynamic Schottky Diode (DSD) generators need to be undertaken.

**Figure 1:**
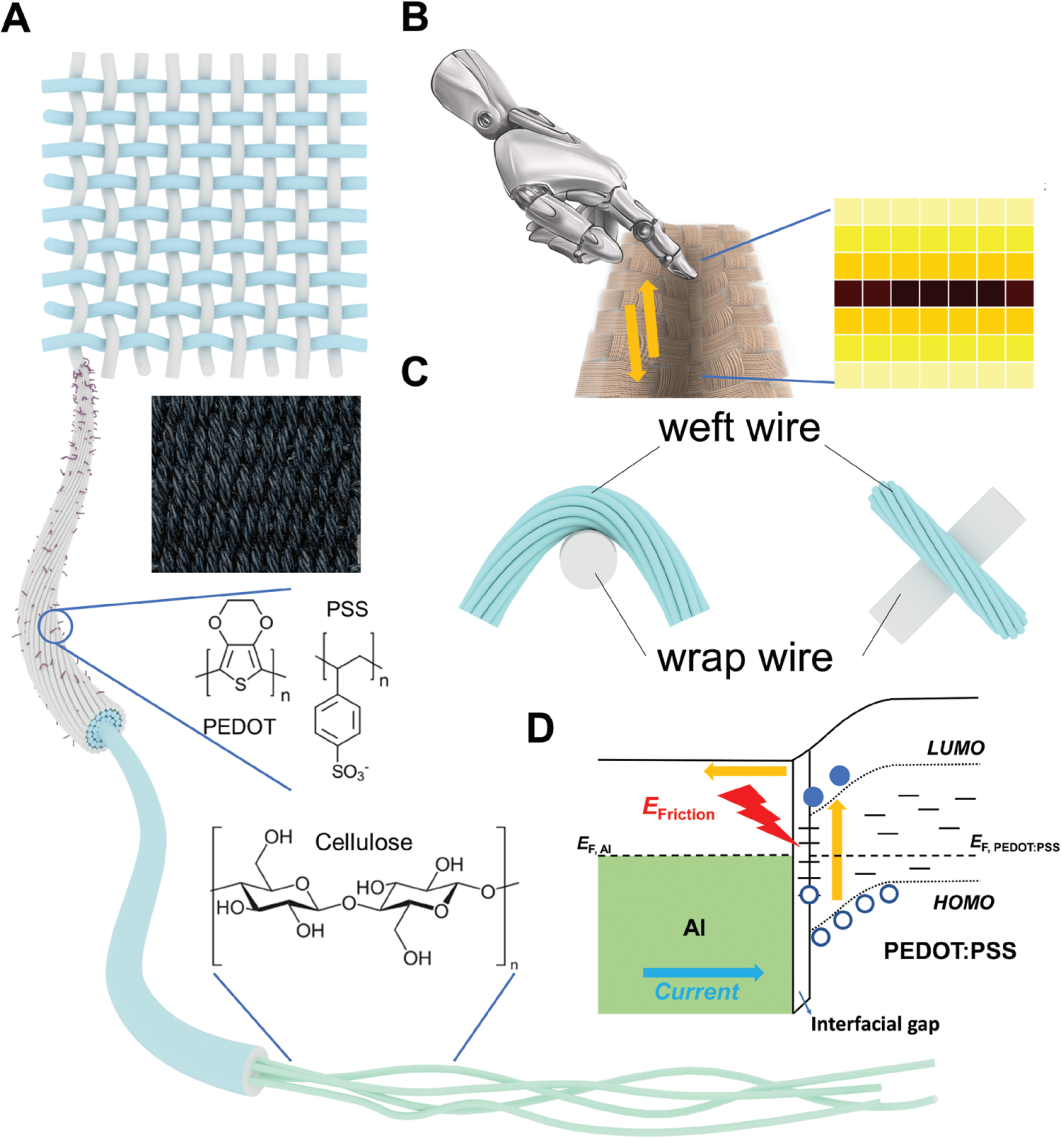
Self‐powered Dynamic Schottky Diodes‐based Smart Textile. Schematic of A) the hierarchical structure of cellulose textile. B) Biaxial motions detection and sensing networks with the smart textile. C) A representative thread junction acting as a Schottky diode. D) Energy band diagram of the DC generation in Dynamic Schottky Diode under short‐circuit condition.

Here, we demonstrate a self‐powered, highly efficient cellulose‐based smart textile multifunctional sensor via integrating a novel DSD generator with cellulose textile and their on‐design woven structure. In particular, with desirable comfort, durability, and hierarchical structure, cellulose fibers are used as the multiscale scaffold and integrated with Poly (3,4‐ethylene dioxythiophene): poly (styrene sulfonate) (PEDOT: PSS), the intrinsically conductive polymer, to fabricate the composite fibers aiming for DC power generation and providing sensing capability (**Figure** **1**A). Based on the dynamic motion working mode, biaxial motions in normal and tangential directions that trigger the kinetic energy‐electricity conversion of the device could be detected in the form of signal output (Figure 1B). Each junction of the smart textile acts as a Schottky diode (Figure 1C), forming a woven matrix that provides several intersections simultaneously. In detail, a metal‐semiconductor Schottky diode is formed between a metal and p‐type semiconductor that creates a barrier and a depletion layer. Transfer of holes occurs along the established electric potential between the mismatched surface energy levels of the semiconductor and the electrode until a thermodynamic equilibrium is reached with the alignment of Fermi energy levels. At a dynamic contact interface, mechanical impacts such as friction and pulsed pressure are exerted, resulting in a non‐equilibrium carrier transport, which can then be detected in the outer circuit with generated open‐circuit voltage and short‐circuit current (Figure 1D). Moreover, a mitigation strategy with oil‐sealing and interfacial engineering is introduced to address the short lifespan caused by the decaying (e.g., mechanical and electrochemical loss). Featuring excellent performance merits in sensing and wearability, our device, utilizing sustained design and naturally‐occurred hierarchical structured cellulose fibers, exhibits excellent flexibility and stretchability, air permeability, and compatibility with actual clothing, shoes, carpet, etc., marking it as a promising approach to generate electricity from the mechanical motions and serve as a multifunctional sensing network in daily life.

## 2 Results and Discussion

### 2.1 Material Characterization: Fiber & Textile

Cellulose‐based fibers are considered a promising raw material for smart textiles due to their flexibility, wearability, comfort, and washability. Cellulose fibers are featured with a hierarchical structure from the nano‐ to the macro‐scale, providing scaffolds for hosting functional materials. To enhance the wearability and practicability of cellulose fibers, they are usually twisted into thread or further woven into the textile.

**Figure 2:**
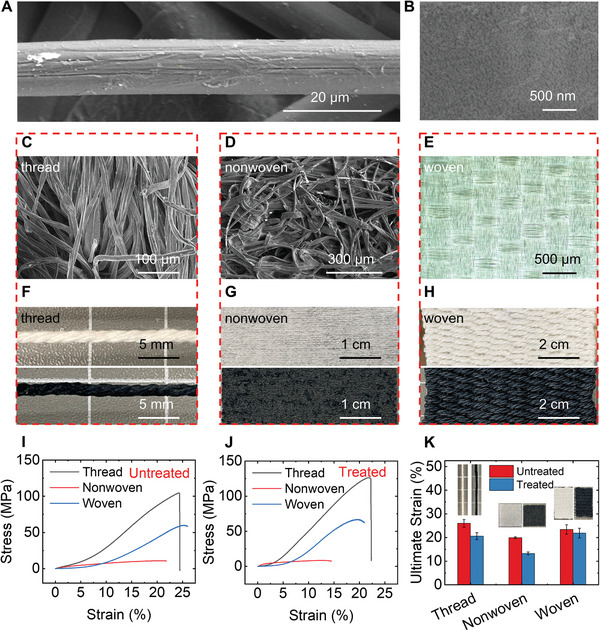
Material characterization: fiber & textile. A) SEM image of a single cellulose fiber. B) SEM image of the cross‐section of cellulose fiber. C) SEM image of twisting threads. D) SEM image of the nonwoven textile. E) Optical image of the woven textile (nylon). F–H) Optical images of thread F), nonwoven textile G), and woven textile H) before (upper) and after (lower) being coated with PEDOT: PSS. I–J) Tensile tests of thread, nonwoven textile, and woven textile without (I) & with (J) coating PEDOT: PSS. K) Ultimate strains for PEDOT: PSS treated and untreated fibers, nonwoven textiles, and woven textiles.

**Figure** **2**A shows well‐oriented micro‐sized single fibers that are naturally aligned with a diameter of tens of micrometers. The cross‐section of a single fiber is shown in Figure 2B, where a number of nanofibers are within a single fiber's size down to the nanoscale. This hierarchical structure provides a multiscale scaffold for hosting functional materials. The scanning electron microscope (SEM) images of the thread, nonwoven textile, and woven textile are shown in Figures 2C–E, respectively. It can be seen that the thread (Figure 2C) consists of plentiful twisted cellulose fibers. At the same time, the nonwoven textile (Figure 2D) is made up of crushed cellulose fibers forming a randomly established network. The woven structure (nylon as an example) shown in Figure 2E provides a unique pattern, where warp wires and weft wires form a natural matrix containing multiple junctions. Typically, the threads are twisted first and then woven into the textile with this structure, which is also utilized in this paper.

To form the DSD system using cellulose fibers, PEDOT: PSS, one of the most universally used organic semiconductors which have great adhesion with cellulose fiber due to its hydrophilicity and flexibility,^[^ ^32^, ^33^ ^]^ is chosen in this work. The thread was cut into 3.94 inches (10 cm) each in length, while the nonwoven textile was cut to the size of 2 inches × 2 inches. Conductive ink was prepared by mixing 95 vol.% PEDOT: PSS (1.3 wt.% dispersion in H_2_O, conductive grade) and 5 vol.% Dimethyl sulfoxides (DMSO) (Hybri‐MaxTM, sterile‐filtered, BioReagent, suitable for hybridoma, ≥99.7%) together. The cotton threads were then transferred into 50 mL conductive ink in vials. A vacuum oven with a pressure of 25 Pa was used to assist in the infiltration of the ink into the multiscale scaffold of cotton thread. The infiltration process was completed after around one hour when no additional increase of the ink in the cotton thread can be observed. After infiltration, the samples were removed from the vials and dried using an air‐forced convection oven at 100 °C for 15 min. Each nonwoven textile (2 inches × 2 inches) was mixed with 5 mL conductive ink following the same infiltration and drying process. The woven textile was woven with conductive threads using a weaving machine. Figure 2F–H shows optical images of the thread, the nonwoven textile, and the woven textile before and after treatment of PEDOT: PSS. The color change was observed after the treatment, while the energy dispersive X‐ray analysis (EDX) element maps (Figures S3–S4, Supporting Information) of carbon (C) and sulfur (S) provided further verification. The characteristic element S that comes from PEDOT: PSS represents the successful combination of cellulose fiber and PEDOT: PSS.

To further investigate the influence of adding PEDOT: PSS, tensile tests of untreated (Figure 2I) and treated (Figure 2J) thread, nonwoven textile, and woven textile are conducted. In all untreated samples, thread features the highest strength of 103 MPa and Young's modulus of 497 MPa. Due to the unaligned structure and randomly formed network of cellulose fibers, nonwoven textile has the lowest strength of 10 MPa and Young's modulus of 47.7 MPa, showing softer and more flexible performance. After the treatment of PEDOT: PSS, the strength and Young's modulus of thread is increased to 126 MPa and 700 MPa, respectively, and Young's modulus of woven textile is increased from 277 MPa to 501 MPa. However, all three types of samples present lower ultimate strains after being coated with PEDOT: PSS (Figure 2K), revealing more brittle behaviors. The existence of PEDOT: PSS that fills up the void spaces among cellulose fibers could account for the brittle phenomenon. However, as a tradeoff of forming DSD, the mechanical properties still satisfy the requirement of wearable devices in daily life.

### 2.2 Interfacial Mechanism and Characterizations of the DC Output

To address the long‐lasting output decay problem, we found that two mechanisms may influence the lifespan of the system that occurred at the metal‐organic interfaces: mechanical loss from frictions and electrochemical (EC) loss from electrochemical reactions. The decay problems that shorten the lifespan of the DC generator and organic‐metal contact have been reported in previous works.^[^ ^34^, ^35^ ^]^ Accordingly, we have developed solutions to increase the lifespan of the DSD system in two ways: (1) utilizing an oil‐sealing strategy to decrease both mechanical loss and EC loss, and (2) applying the identical counter‐electrodes to reduce the potential interfacial EC reaction. Our results show that both EC and mechanical loss can be successfully mitigated, resulting in a longer lifespan for the proposed smart textile system.

In this work, PEDOT: PSS has work function Φ_PEDOT: PSS_ ≈5.0 eV, to enlarge the signal outputs, aluminum (Al) (Φ_Al_ = 4.06–4.26 eV) is selected as the working electrode for the mechanical‐electrical conversion.^[^ ^29^ ^]^ Figure S39 (Supporting Information) shows that the Al‐Al pair has the largest output signal compared with the Ni‐Ni and Zn‐Zn pairs, when used in conjunction with PEDOT: PSS. With the existence of oil, the friction in the interface gets decreased, and less damage to the PEDOT: PSS is observed, indicating a reduced material loss due to mechanical force. Meanwhile, the addition of oil prevents the absorption of moisture by the textile and therefore can mitigate the EC loss in wet conditions. As a result, the sample with the oil‐sealing strategy could show stabilized outputs.

**Figure 3:**
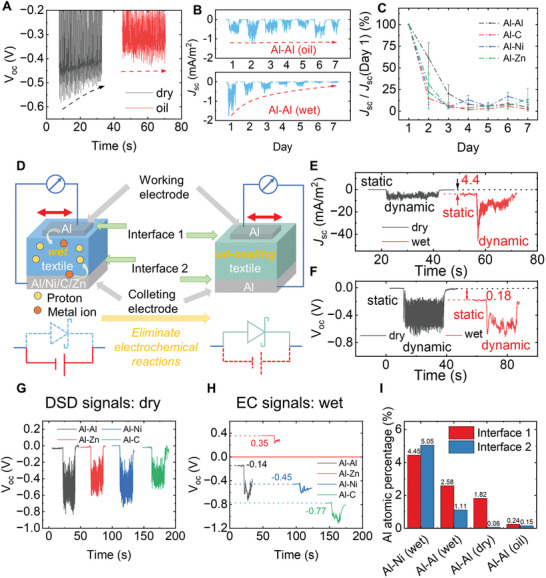
Interfacial mechanism and characterizations of the DC output. A) Comparison of outputs decaying over time in dry and oil‐sealed smart textiles. B) Interval cycling performance comparison of oil‐sealed sample and sample under wet conditions. C) Comparison of interval cycling performance showing decaying of DSD outputs (*J*_SC_). D) Schematic mechanism of electrochemical decay and oil‐sealing strategy. E,F) Different kinds of E) short‐circuit current density (*J*_SC_) and F) open‐circuit voltage (*V*_OC_) output of Al‐Al pair under different moisture conditions (wet and dry) and motion conditions (static and dynamic). G) Comparison of DSD signals in different pairs of electrodes. H) Comparison of EC signals in different pairs of electrodes. I) Al atomic percentage at interface 1&2 of electrode pairs under different conditions.

As is shown in **Figure** **3**A, the *V*_OC_ of the unsealed sample (dry) shows an obvious decay from ‐0.58 to ‐0.46 V after ≈20 s. This initial decay comes from the friction between the electrode and textile, which is considered the material loss from the mechanical force. However, the sample with paraffin oil sealing strategy (oil) features stable open‐circuit voltage outputs (*V*_OC_) ranging ≈0.41±0.02 V, indicating the effective mitigation of the mechanical loss. In order to investigate the influence of EC loss, interval cycling, which records the DSD outputs after being short‐circuited for 24 h, is carried out. It is shown in Figure 3B that the oil‐sealing sample maintains a high short‐circuit current density (*J*_SC_) ≈ 0.7 mAm⁻^2^ even after 7 days (144 h). In contrast, the output of the sample with the existence of moisture (wet) displays a sharp reduction of 97%, dropping from ‐1.7 mA m⁻^2^ to ≈0.04 mA m⁻^2^ after a 7‐day decay. Figure 3C shows the change in the percentage of current outputs to the initial output in different pairs in interval cycling. Different conductors (Al/Ni/Zn/C) are introduced to the collecting electrodes. It is shown that the current density outputs of devices decaying with water have a sharp drop of 90% after a 4‐day (96 h) decay, and less than 10% remains after 7 days. Among all current collectors in pairs of Al, the pair of the same Al collecting electrode shows a moderate drop (40%) of current outputs resulting from a less intensive reaction in the first 24 h. The decay results from the electrochemical reactions under wet conditions between electrodes and PEDOT: PSS, which changes the property of the organic semiconductor and reduces the amount of the effective carriers (Figure S8, Supporting Information). Consequently, EC and mechanical losses should be diminished in pursuit of long‐lasting, high‐performance DSD devices.

In general, the Schottky Diode performance is the dominant power generation effect under dry conditions. However, PEDOT: PSS and cellulose fibers are hydrophilic and can absorb moisture from the ambient environment, resulting in humidity conditions in textiles. Consequently, the proton and PSS^−^ will provide an ionic environment for the electrochemical reaction (Figure 3D). Therefore, under wet conditions, the electrochemical effect emerges and distinguishes itself from the Dynamic Schottky Diode (DSD) power generation. In these cases, the working and collecting electrodes serve as a pair of positive and negative electrodes in a primary cell. Such a hypothesis is verified by electrochemical impedance spectroscopy (EIS) (Figure S6, Supporting Information) and moisture‐dependent signals (Figure S7, Supporting Information). It is shown in Figure S6 (Supporting Information) that, under wet conditions, the ion diffusion emerges under the low‐frequency range, providing an ionic and electrolyte environment for EC reactions, while such diffusion is not found under dry conditions. Meanwhile, the static outputs show a positive correlation with the moisture content (Figure S7, Supporting Information), indicating a response originating from the moisture‐dependent ionic activities rather than the moisture‐independent electronic conduction.

To explicitly figure out the influence of electrochemical behavior and DSD generator behavior, different signals have been identified under different moisture conditions (wet and dry) and motion‐trigger conditions (static and dynamic). *J*_SC_ and *V*_OC_ under various conditions of the Al‐Al electrode pair are shown in Figure 3E,F. The signals under the dry&static condition are recognized as Static Schottky Diode (SSD) signals. Compared to signals under different conditions, SSD signals are negligible, which can be considered the zero baseline of all outputs. When sliding motion is applied under dynamic conditions, mechanical‐electrical conversion occurs; thus, signals under the dry&dynamic condition are recognized as the DSD generator signals. After the status changes from static to dynamic, the *J*_SC_ arises from ‐0.1 mA m⁻^2^ to ‐8.9 mA m⁻^2^ (Figure 3E), and *V*_OC_ emerges from ‐0.01 to ‐6.5 V (Figure 3F). When returning to the static conditions, both *J*_SC_ and *V*_OC_ reset again. Compared to the SSD signals, significant increases under the wet&static condition in *J*_SC_ (4.4 mA m⁻^2^) and *V*_OC_ (0.18 V) have been observed, which are identified as the electrochemical (EC) signals resulting from the reaction at the interface. Unlike the DSD signals, the signals under the wet&dynamic condition feature a less periodic tendency toward motion applied and have more obvious fluctuation, indicating inconsistency with the motion frequency. With the existence of moisture, the outputs are more susceptible to the reaction rate of the self‐assembly primary cell. They, therefore, are affected by various parameters like contact area and motion velocity.

Comparisons of the pairs of electrodes (Zn/Ni) are shown in Figures S11, S12 (Supporting Information). It is found that EC signals are higher than DSD signals in all pairs of electrodes. Figure 3G presents DSD signals in different pairs of electrodes. With Al acting as the working electrode, the collecting electrode in the same type (Al‐Al) induces symmetric bending of the energy band at the two interfaces, which may account for the higher *V*_OC_ of ≈0.7 V compared with other pairs. EC signals are affected by chemical reaction potential differences between two electrodes, as indicated in Figure 3H. Among all pairs, the Al‐Al pair features the lowest EC outputs of ‐0.14 V, and Al‐C features the highest with ‐0.77 V. Significantly, the Al‐Zn pair shows the opposite output direction to others, also proving EC reactions occurred at the interfaces (Figures S13, S14, Supporting Information).

To investigate the loss mechanism at interfaces, EDX analysis is conducted to measure the reactive atomic percentage (Figure 3I). In the Al‐Ni pair under wet conditions, the reactive atomic percentage of Al is measured to be 4.45%, which is much higher than that in the Al‐Al pair (2.58%), indicating a more intensive reaction and consumption of electrode materials. Besides, the reactive Al element is even found in interface 2, although there is no direct contact between the Al working electrode and interface 2. This could be caused by the movement of reactive Al^3+^ from the working electrode. Compared to wet conditions where the EC reaction occurs, the Al‐Al pair shows less Al consumption under dry conditions. In this case, the observed Al comes from the mechanical loss happening at interface 1, and seldom Al is found at interface 2 because of the less relative displacement between the textile and collecting electrode. Moreover, the minor Al element (0.24%) is located in the oil‐sealing pair. Here, it can be inferred that both mechanical and EC loss are reduced; thus, less electrode material is consumed in the generator.

Therefore, aiming at extending the lifespan of smart textiles, the oil‐sealing strategy is proven to be an effective method to significantly control the humidity and reduce both EC and mechanical loss. Additionally, identical counter‐electrodes are utilized to minimize the potential interfacial EC reaction. Our results show that both EC and mechanical loss can be successfully mitigated, resulting in a longer lifespan for the proposed smart textile system.

### 2.3 Interfacial Mechanism and Characterizations of the DC Output

**Figure 4:**
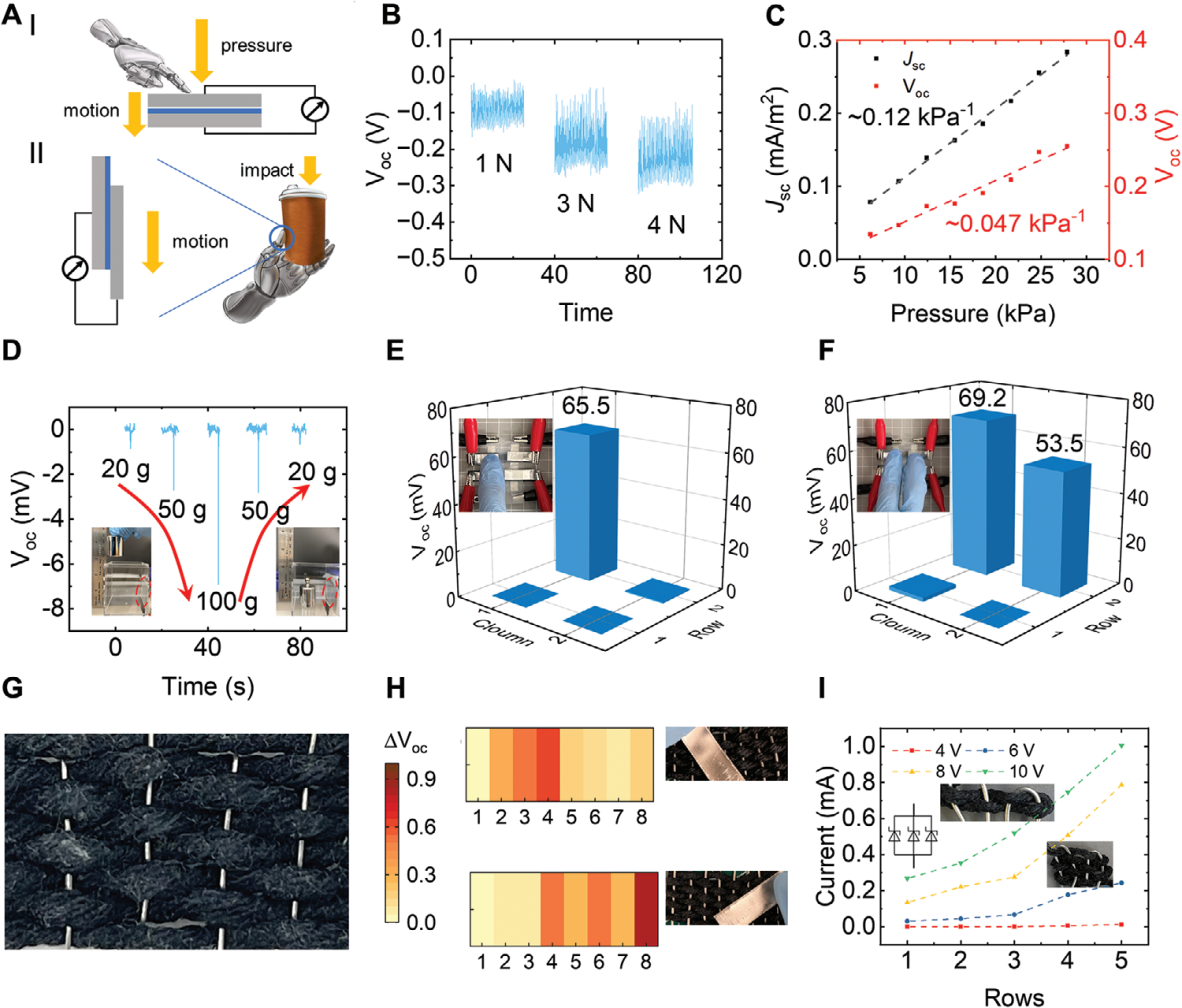
Demonstration and characterizations of biaxial sensing networks. A) Schematic of the scenario of smart textile for detecting normal pressure (I) and shear motion (II). B) Voltage outputs of sliding motion under different normal pressure. C) Characterizations of pressure‐dependent sensors in terms of voltage and current outputs. D) Measurement and photos of voltage outputs in response to mechanical impact. E–F) Measurement of voltage outputs in pressure sensor matrix under 1‐junction (E) and 2‐junction (F) conditions. G) Photo of woven structure in smart textile. H) Demonstration of the motion sensing array of different distributions. I) Effective in‐parallel circuit of woven structure and its output as a function of the number of rows.

Motion trigging and detection is an essential topic in wearable devices due to its broad application scenarios.^[^ ^36^, ^37^, ^38^ ^]^ However, many intricate networks of sensors are designed only in response to one type of deformation. As indicated in **Figure** **4**A, we use DSD‐based smart textiles to demonstrate a biaxial motion sensor that could detect both normal pressure (I) and shear motion (II). With different pressures applied to the working collector, the outputs of the oil‐sealing device that is triggered by the dynamic motion exhibit different pressure‐related amplitudes. It is shown in Figure 4B that DSD signals increase with the increase of applied pressure. Figure 4C shows the linear relationship of *J*_SC_ and *V*_OC_ in response to pressure applied, and the sensitivity is calculated as 0.12 kPa⁻^1^ and 0.047 kPa⁻^1^, respectively, according to the following equations:

(1) $$\frac{\Delta J}{J_{i}}=\frac{\left(J-J_{i}\right)}{J_{i}}\;\;\mathrm{or}\;\;\frac{\Delta V}{V_{i}}=\frac{\left(V-V_{i}\right)}{V_{i}}$$

(2) $$S=\frac{\delta \left(\frac{\Delta J}{J_{i}}\right)}{\delta P}\;or\;S=\frac{\delta \left(\frac{\Delta V}{V_{i}}\right)}{\delta P}$$

where *J*_i_ and *J*, *V*_i_ and *V* refer to the short‐circuit current density or open‐circuit voltage at the initial state and after pressure loading, and *P* indicates the loading pressure on the sensor, respectively.

Beyond detecting the normal pressure, the sensor is also sensitive to the shear motion produced by sliding the working electrode. This effect could be applied to detecting the vertical impact that happens when the mechanical limb lifts a weighted object, as shown in Figure 4A II. Meanwhile, smart textiles provide excellent flexibility and conformality, easily fitting into complex geometries that mimic mechanical limbs. As proof of concept, the impact sensor is demonstrated to detect the mechanical impact caused by different weights of objects dropping from the same height (80 mm), leading to the sliding of the working electrode and the signal outputs. As is shown in Figure 4D, the cube box is fixed by the calipers, and smart textile is attached between the box and one side of the calipers (in the red dashed circle). The *V*_OC_ is measured to be ‐0.84, ‐2.7, and ‐6.9 mV with dropping masses of 20, 50, and 100 g, respectively, which shows a proportional relationship between mass and signal output. This demonstration indicates the potential of detecting biaxial force in human‐machine interactions.

Furthermore, inspired by the woven structure, a 2 × 2 matrix is set up to detect different pressure distributions. As is shown in Figure 4E,F, the signal output responds to the loading pressure correspondingly. Specifically, when only one finger presses the first junction, the signal outputs at that junction emerge as 65.5 mV while the others remain in “silence” states (<0.1 mV). When two fingers press the two junctions at the same row, the corresponding two junctions show emerging signal outputs of 69.2 and 53.5 mV. It is proved that the results are susceptible to the distribution of pressure, which provides the potential for the recognition of object distribution.

Moreover, when conductive threads and metal wires are woven together, acting as wrap wires and weft wires severally, metal‐semiconductor interfaces are scaled up, forming a matrix of Schottky Diodes (Figure 4G). Therefore, our DSD‐based smart textile can detect sliding motion in the tangential direction as a sensing array. Figure 4H shows the sensing array in response to the applied sliding motion. The color contrast corresponds to the electromechanical coupling intensity: the darker color indicates a more considerable voltage change due to the heavier sliding motion upon them. In comparison, the lighter color displays a minor voltage change for the light loadings. It can be seen in Figure 4H that when sliding motion is applied to rows #2‐4, the corresponding arrays feature a more significant voltage change compared to other arrays without sliding motion. This variation implies the sensor arrays' spatial recognition ability and immense potential for motion distribution detection.

Besides, with the diode matrix provided by the woven structure, a circuit of multiple diodes can be created with the overall output being amplified. As is shown in Figure 4I, a conductive thread and an aluminum wire are woven together, forming an in‐parallel circuit consisting of several diodes. Each row of the conductive thread forms four contact points (4 diodes) with the aluminum wire. As the number of conductive threads increases, the diodes of the in‐parallel circuit will increase, leading to signal amplification. With different voltages applied between the anode (conductive wire) and cathode (metal wire), the current that passes through the circuit increases with the number of rows. It is shown in Figure 4I that, with a voltage of 10 V applied at two sides, the number of rows of wrapping conductive wires increases from 1 to 5, and the signals increase from 0.27 to 1 mV accordingly. In general, we have shown that our smart textile can serve as a self‐powered sensing network without an additional power supply, which detects motion from both normal and tangential directions, proving the broad applications of smart textiles in human‐machine interactions.

## 3 Conclusion

In this work, we have developed a smart textile integrated with self‐powered sensing networks, which features excellent stretchability, bendability, washability, and comfort, without additional uncomfortable, bulky, and rigid power sources. Via a facile infiltration process, an active polymer‐based semiconductor is incorporated into the primary thread and textile toward the realization of a high‐performance, self‐powered biaxial motion detection, and sensing network. The thread behaves as electronically conductive wires, while the interconnections can be achieved by simple weaving. With the woven structure's formation of diodes in parallel circuits, the signal outputs are amplified by design. In addition, two decaying mechanisms at the organic‐metal interface have been identified. Mitigation strategies based on oil‐sealing and identical counter‐electrodes have been proposed to extend lifespan. Therefore, both the technological obstacle in integrating sensing networks into primary smart textiles and the material limitations in incorporating performance merits with mechanical deformability and durability are overcome by building DSD in smart textiles. It has also been proven that smart textile provides an ideal platform for spatial pressure and shear motion distribution recognition. The proposed device opens new opportunities for the development of wearable electronics in next‐generation smart textiles, enabling techniques to build sensor systems that can be comfortably integrated into primary clothing, shoes, carpet, etc.

## 4 Experimental Section

### Morphology Characterization of Conductive Thread, Nonwoven Textile, and Woven Textile

Scanning electron microscopy (SEM) and energy dispersive X‐ray analysis (EDX) were conducted on Helios G4 UX dual‐beam equipment to observe the morphology and the hierarchical structures.

### Tensile Tests of Conductive Thread, Nonwoven Textile, and Woven Textile

Tensile tests were conducted using ADMET Load Frame as a tensile meter to determine the stress‐strain curves for threads, nonwoven textiles, and woven textiles respectively. To better clamp the samples, sandpaper was attached between the samples and the transducer. Before each test, the initial length and cross‐section were measured. Forces were applied along the weft direction in woven textiles. The representative curve in each type is shown in Figure 2I. The error bars and average values of ultimate strains were derived based on the test results of three samples, shown in Figure 2K. The characterization processes were the same for all samples (Figure 2J).

### Electrochemical Characterizations of Conductive Thread, Nonwoven Textile, and Woven Textile

Electrochemical characteristics were assessed on VSP Biologic electrochemical workstation, and the electrochemical performance was measured with Ag/AgCl electrodes. Electrochemical impedance spectroscopy (EIS) measurement was conducted for every condition with a frequency from 100 Hz to 1 MHz.

### Signal Output Measurements

The output measurements included open‐circuit voltage (*V*_OC_) and short‐circuit current density (*J*_SC_). Each sample was annealed on the hot plate at 100 °C for 15 min before the measurement, then applied with different moisture or oil conditions. At room temperature, the test was performed using a multimeter (DAQ6510, Keithley). According to the treatment after annealing, the measurement can be divided into dry (without adding DI water or paraffin oil), wet (adding 200 µL DI water per inch^2^), and oil (adding 200 µL paraffin oil per inch^2^) conditions. Whether the motion is applied on the working electrode or not, the measurements can be identified as static (working electrode kept static) and dynamic (working electrode moved with applied motions) conditions. Moisture‐dependent tests were conducted under static conditions, and the moisture content was controlled by adding different volumes of DI water. Each point of decaying testing (interval cycling) was conducted after the sample was short‐circuited for 24 h. Continuous cycling was conducted on the set up cycling platform; each cycle's displacement was 10 mm, and the velocity was 15 mm s⁻^1^. Pressure‐dependent tests were also performed on the platform with a displacement of 10 mm and a velocity of 51 mm s⁻^1^ for each cycle. Impact‐dependent tests were demonstrated according to the different mass dropping from a height of 80 mm. The signals from the 2×2 matrix were recorded by a portable multimeter and responded to the finger pressure. The signal superposition tests were performed using a multimeter (DAQ2450, Keithley) for sweeping the C‐V curve, and voltage was applied from ‐10 to 10 V at the two terminals at each test. The signal outputs of distribution array tests were recorded using the PXI system (PXIe‐1082 Chassis, PXIe‐8840 Controller, and PXIe‐4302 Modules, National Instruments).

## Conflict of Interest

The authors declare no conflict of interest.

## Supporting information

Supporting Information

Supporting Information Movie 1

Supporting Information Movie 2

Supporting Information Movie 3

## Data Availability

The data that support the findings of this study are available from the corresponding author upon reasonable request.
