# Supplementary material for: Self‐Powered Smart Textile Based on Dynamic Schottky Diode for Human‐Machine Interactions
Source: Adv Sci (Weinh). 2023 Feb 13;10(11):2207298. doi: 10.1002/advs.202207298 (PMC10104626; doi:10.1002/advs.202207298)
Supplement: Supplementary file 1 — Supporting Information [file ADVS-10-2207298-s003.pdf]

Supporting Information

Self-powered Smart Textile based on Dynamic Schottky Diode for Human-Machine Interactions

Pengfei Deng,[1] Yanbin Wang,[1] Ruizhe Yang,[2,3] Zijian He,[1] Yuanqiu Tan,[4] Zhihong Chen,[4] Jun Liu,[2,3] and Tian Li[1]**

This PDF file includes:

Figure S1. Schematic of mechanism of dynamic Schottky diode
Figure S2. Signal output of one-junction organic-metal contact
Figure S3. SEM and EDX mapping of composite thread coated with PEDOT: PSS
Figure S4. SEM and EDX mapping of nonwoven textile coated with PEDOT: PSS
Figure S5. Long-term continues cycling between samples with and without oil-isolation
Figure S6. EIS of samples under dry and wet conditions
Figure S7. Comparison of moisture content dependent *Jsc* and *Voc* EC signals in different pairs
Figure S8. Mechanism of EC reaction and hydrophilicity change after decaying
Figure S9. Hydrophilicity of textile before decaying
Figure S10. Hydrophobicity of textile after decaying
Figure S11. Comparison of 4 types of signals outputs in Al-Zn pair
Figure S12. Comparison of 4 types of signals outputs in Al-Ni pair
Figure S13. EC signals of Zn in pair of polished Al (Al_2O_3)
Figure S14. EC signals of Zn in pair of finished Al
Figure S15. SEM of textile interface 1 in Al-Al (dry) pair
Figure S16. EDX mapping of textile interface 1 in Al-Al (dry) pair
Figure S17. EDX spectrum of textile interface 1 in Al-Al (dry) pair
Figure S18. SEM of textile interface 2 in Al-Al (dry) pair
Figure S19. EDX mapping of textile interface 2 in Al-Al (dry) pair
Figure S20. EDX spectrum of textile interface 2 in Al-Al (dry) pair
Figure S21. SEM of textile interface 1 in Al-Al (wet) pair
Figure S22. EDX mapping of textile interface 1 in Al-Al (wet) pair
Figure S23. EDX spectrum of textile interface 1 in Al-Al (wet) pair
Figure S24. SEM of textile interface 2 in Al-Al (wet) pair
Figure S25. EDX mapping of textile interface 2 in Al-Al (wet) pair
Figure S26. EDX spectrum of textile interface 2 in Al-Al (wet) pair
Figure S27. SEM of textile interface 1 in Al- Ni (wet) pair
Figure S28. EDX mapping of textile interface 1 in Al-Ni (wet) pair
Figure S29. EDX spectrum of textile interface 1 in Al- Ni (wet) pair
Figure S30. SEM of textile interface 2 in Al-Ni (wet) pair
Figure S31. EDX mapping of textile interface 2 in Al-Ni (wet) pair
Figure S32. EDX spectrum of textile interface 2 in Al- Ni (wet) pair
Figure S33. SEM of textile interface 1 in Al-Zn (wet) pair

Figure S34. EDX mapping of textile interface 1 in Al- Zn (wet) pair
Figure S35. EDX spectrum of textile interface 1 in Al- Zn (wet) pair
Figure S36. SEM of textile interface 2 in Al- Zn (wet) pair
Figure S37. EDX mapping of textile interface 2 in Al- Zn (wet) pair
Figure S38. EDX spectrum of textile interface 2 in Al- Zn (wet) pair
Figure S39. Electrode metal selection
Figure S40. Photos of a nonwoven textile device after washing
Figure S41. Output of DSD signals after washing

Supplementary Text

Mechanism of dynamic Schottky diode

The Schottky diode is formed when metal and semiconductors are in physical contact. A metal-semiconductor junction between a metal and p-type semiconductor creates a barrier or depletion layer. Eventually, electron transfer occurs due to the mismatched surface energy levels when reaching a thermodynamic equilibrium after the alignment of Fermi energy levels at two sides. At a dynamic contact interface, mechanical impacts such as friction and pulsed pressure are exerted, resulting in a non-equilibrium contact material interface and carriers' transition. Finally, the carrier transfer between the interface and movement in the outer circuit leads to the outputs of the open-circuit voltage and short-circuit current.

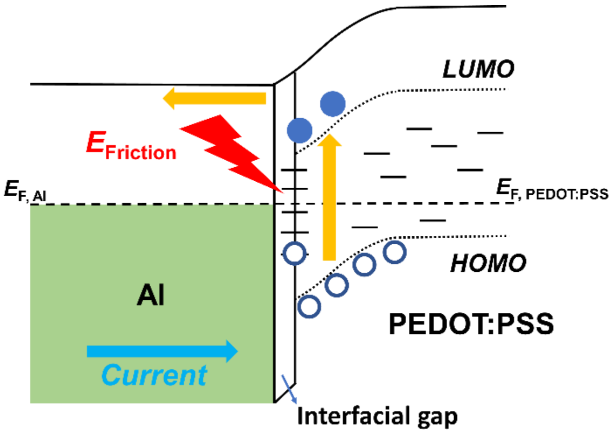

Figure S1. Schematic of mechanism of dynamic Schottky diode

<u>Transient signal of one-junction organic-metal contact</u>

A multimeter (DAQ6510, Keithley) was used to record the signal outputs. Each weft and wrap wire will form a Schottky diode in a woven structure. When mechanical impact like tapping and friction is applied, the dynamic Schottky diode signals will generate. After the motion disappeared, the output returned to zero baseline.

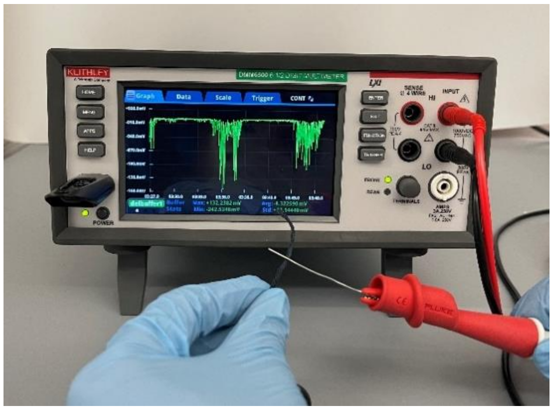

Figure S2. Signal output of one-junction organic-metal contact

SEM and EDX mapping of composite thread, and nonwoven textile

In Figure S3 – S4, carbon and oxygen in EDX mapping come from both cellulose and PEDOT: PSS, while sulfur is identified as the representative of PEDOT: PSS coating. The existence of sulfur proves the deposition of PEDOT: PSS on the thread and nonwoven textile.

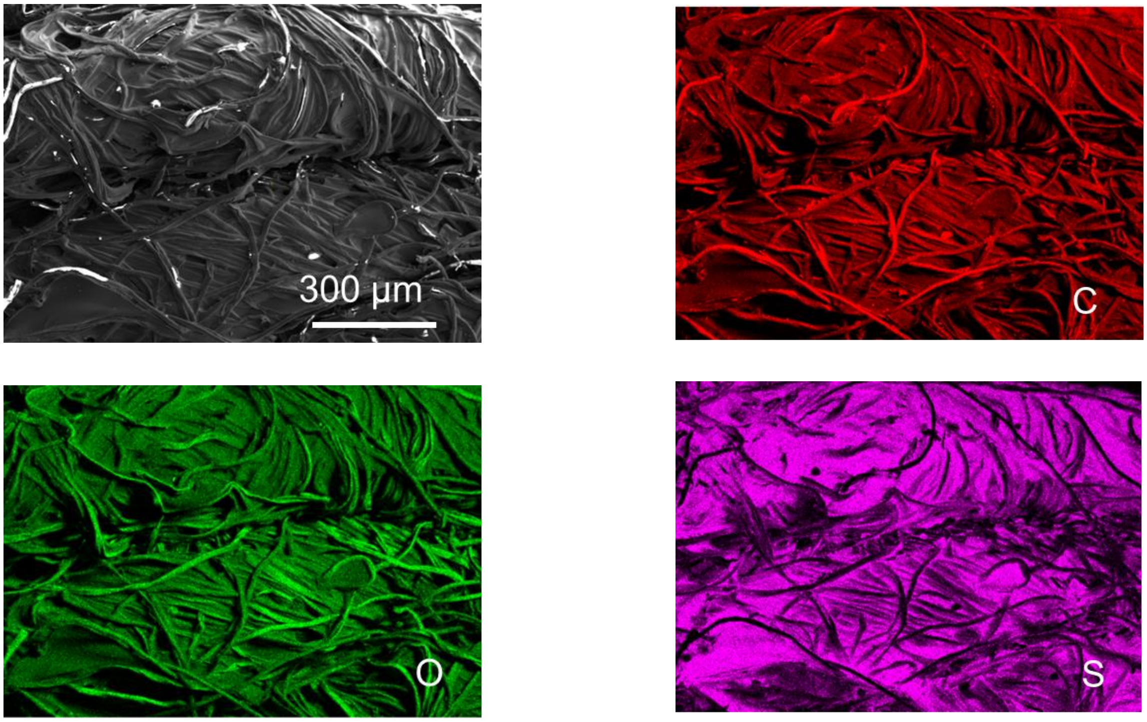

Figure S3. SEM and EDX mapping of composite thread coated with PEDOT: PSS

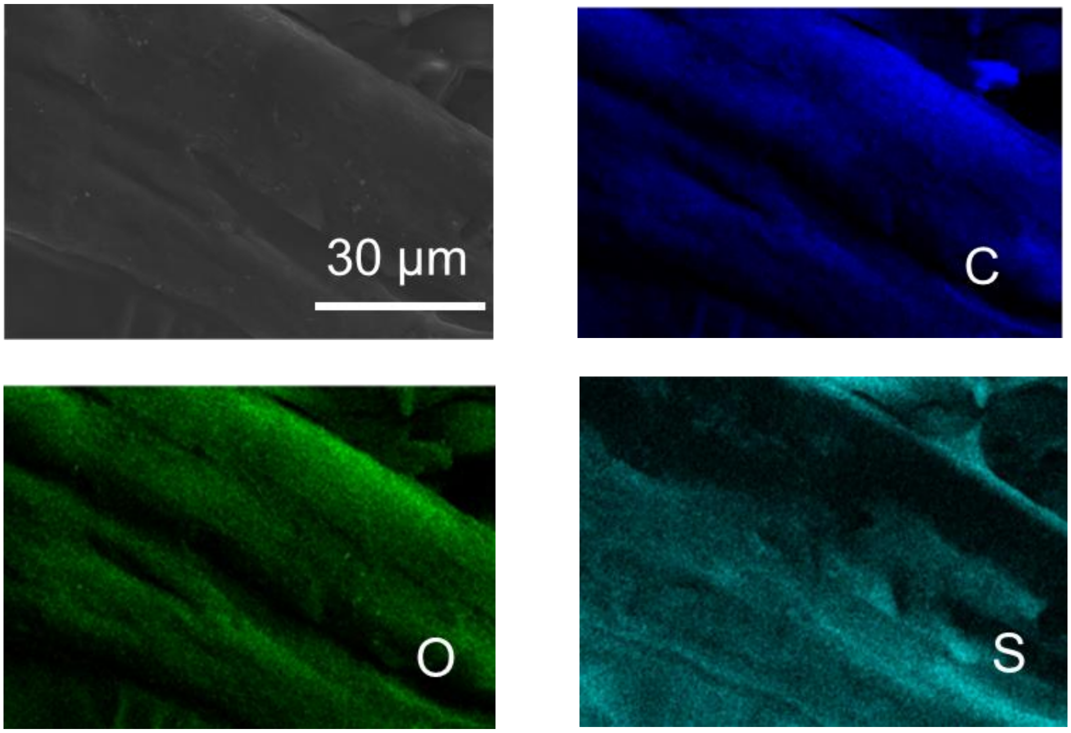

Figure S4. SEM and EDX mapping of nonwoven textile coated with PEDOT: PSS

Long-term continues cycling between samples with and without oil-isolation

Another lifespan testing with continuous cycling is conducted under 1N pressure with/without paraffin oil, aiming to measure mechanical loss. After 5500 cyclings, the open-circuit voltage of the device with paraffin oil decreased from -0.08 V to -0.05 V. In contrast, that of the device without paraffin oil decreased from -0.09 V to -0.02 V. Higher final output has shown in device with paraffin oil, even though it has lower initial output. It's found that the existence of paraffin oil decreases mechanical loss, which is in line with our previous assumption.

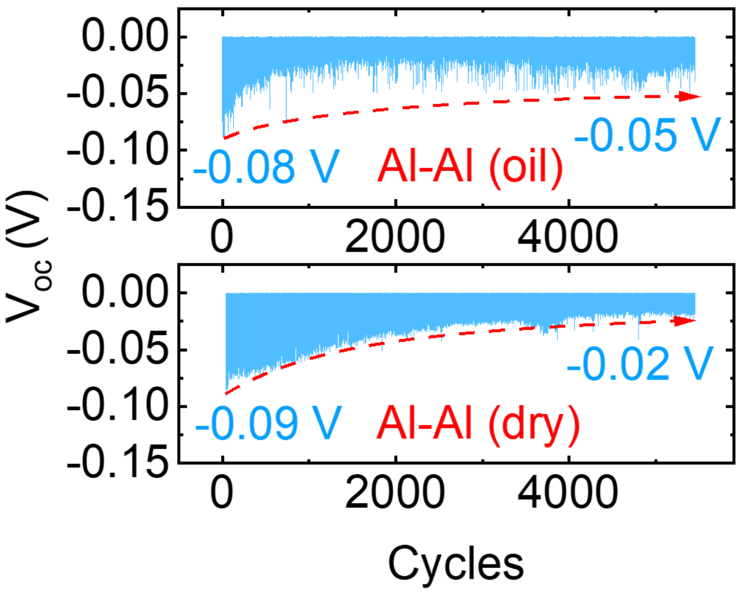

Figure S5. Long-term continues cycling between samples with and without oil-isolation

Moisture-dependent output measurements

EIS measurements of the PEDOT: PSS film under dry and wet conditions are conducted to verify the different ionic and electronic conduction modes. The results indicate that under wet condition, the ionic conductivity emerges as 2.78 S/m (Figure S6). Textile and PEDOT: PSS can absorb water from the ambient environment. To evaluate its impact on performance, we conduct experiments to measure the DSD output signal under static and wet conditions as a function of water content. It is shown in Figure S7 that both static open-circuit voltage (V_{oc}) and short-circuit current density (J_{sc}) respond to the moisture content rapidly. No mechanical motion is applied in a signal generation here. Therefore the signals come from the electrochemical (EC) reactions that occur in the device, and a larger difference in reaction potentials between two electrodes results in larger output signals. Thus, compared with the same Al electrodes at two sides (Al-Al), the Al-Ni pair exhibits higher EC output signals under moist conditions than the Al-Al pair.

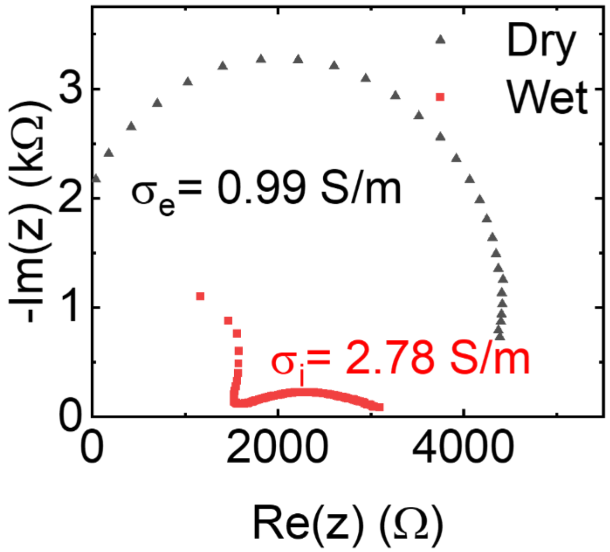

Figure S6. EIS of samples under dry and wet conditions

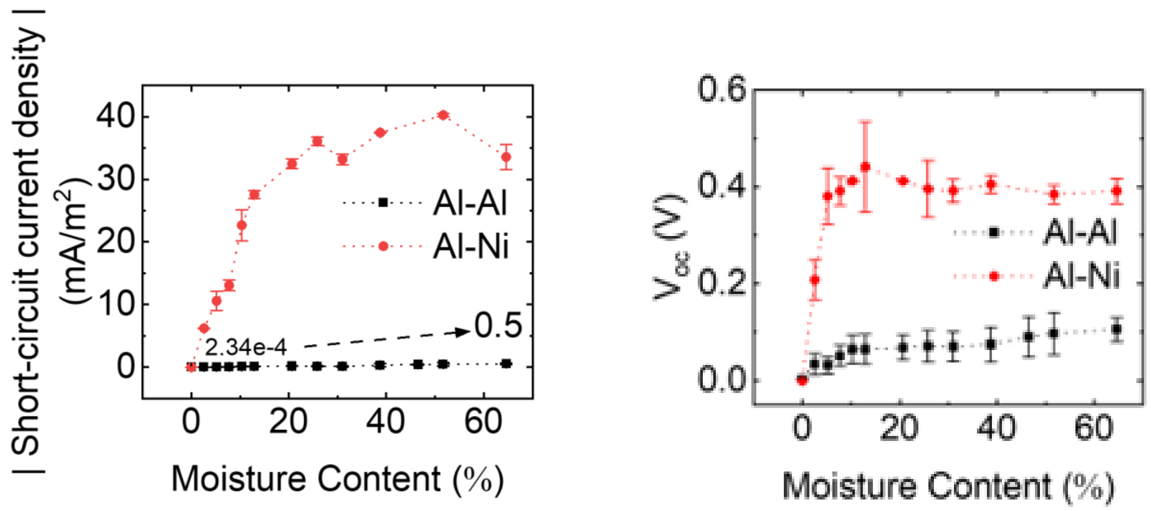

Figure S7. Comparison of moisture content-dependent J_{sc} and V_{oc} EC signals in different pairs

Hydrophobic after decaying

The PEDOT: PSS aqueous solution/dispersion has an excess amount of PSS to enhance the hydrophobic PEDOT particles in water. The interface absorbs water from the environment, and the excess PSSH in the PEDOT: PSS film dissociates into proton and PSS ions again, making the interface conductive. As the PEDOT core is hydrophobic and surrounding PSS is hydrophilic, the cellulose and PEDOT: PSS complex shows hydrophilic properties overall. However, with the electrochemical reaction of metals and PEDOT: PSS at the interfaces, the surrounding PSS^- will combine with metal ions, and PEDOT can be reduced to its neutral state. The first reaction is between protons and the negative electrode, where it consumes negative electrode material. The second reaction is between the negative electrode and PEDOT: PSS [1]:

$$Al - 3e^- \rightarrow Al^{3+} \quad (3)$$

$$Al^{3+} + 3e^- + 3PEDOT^+ + 3PSS^- \rightarrow Al(PSS)_3 + 3PEDOT^0 \quad (4)$$

Consequently, the negatively charged PSS ions detach from the PEDOT chains, which will pair with the metal ions released from the negative electrode. The hole density in the PEDOT chain decreases and the hole transfer is retarded, leading to the decay of output signals (Figure S8). At first, the hydrophilic PSS-rich grain covers the hydrophobic PEDOT-rich grain, making the film appear hydrophilic (Figure S9 & Movie S2). After the possible PEDOT reduction, PSS ions detach from the PEDOT chains and dissolve into the solution, leaving the PEDOT-rich grain on the surface of the film, which appears to be hydrophobic (Figure S10 & Movie S3).

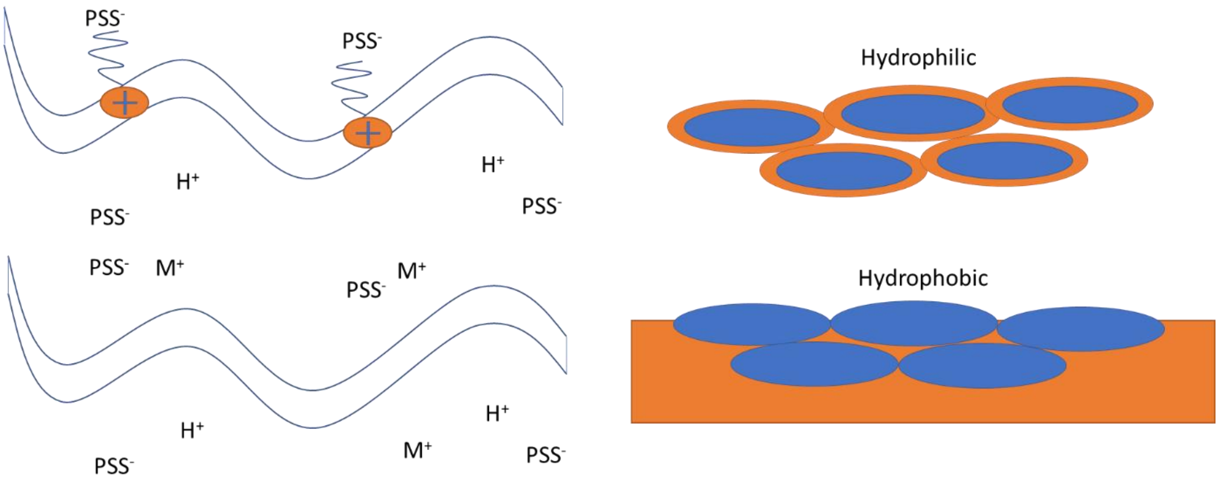

Figure S8. Mechanism of EC reaction and hydrophilicity change after decaying

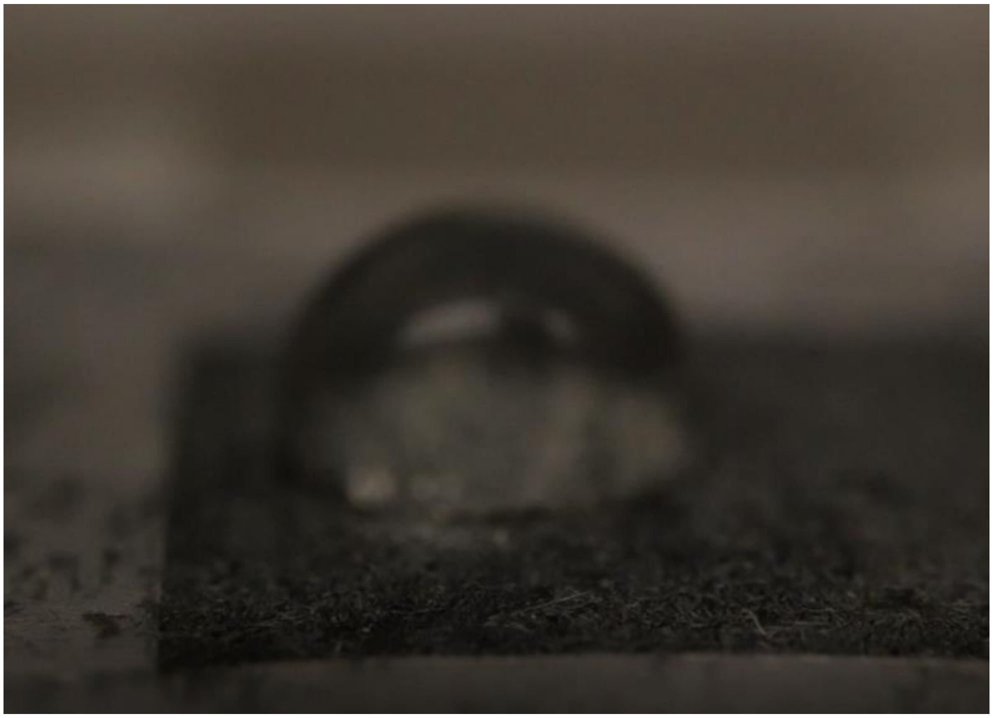

Figure S9. Hydrophilicity of textile before decaying

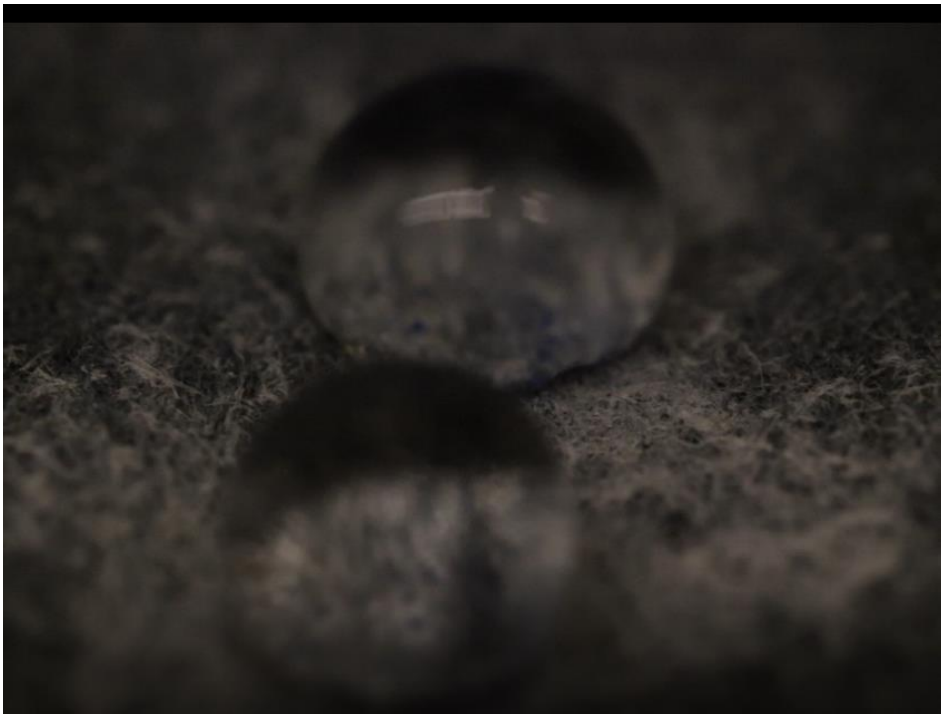

Figure S10. Hydrophobicity of textile after decaying

4 types of signals outputs in different working-collecting electrodes pairs

Four types of signal outputs in different working-collecting electrode pairs (Al-Zn & Al-Ni) are shown in Figures S11-S12. Both pairs show that the signals under wet and static conditions are much higher than that under dry and static conditions, leaving an intensive EC reaction to emerge with the existence of water. It's worth noting that EC signals in the Al-Zn pair feature the opposite direction to others.

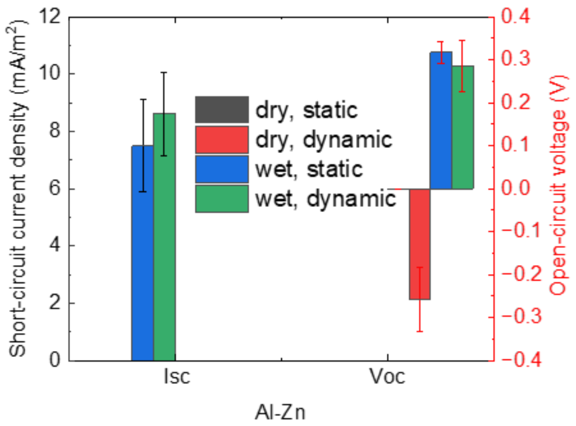

Figure S11. Comparison of 4 types of signals outputs in Al-Zn pair

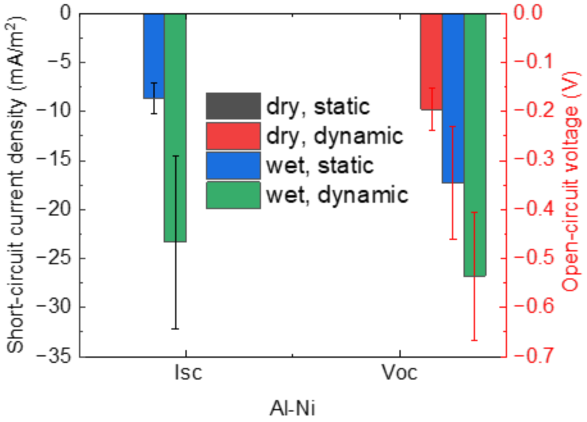

Figure S12. Comparison of 4 types of signals outputs in Al-Ni pair

Zn as collecting electrode

It's well known that the more reactive metal acts as the anode in the primary cell. Although Al is more active than Zn, due to an oxide layer (Al_2O_3) on Al, the Zn will act as an anode compared with less active Al_2O_3. Therefore, a positive direction of signals emerges when the collecting electrode is connected to the negative terminal of the multimeter (Figure S13).

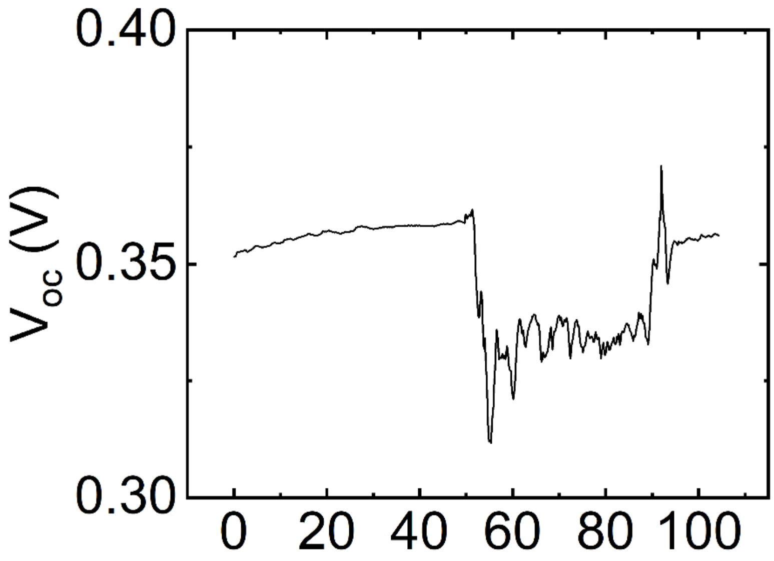

Figure S13. EC signals of Zn in pair of polished Al (Al_2O_3)

However, when the finished Al after removing the oxide layer is used as a working electrode, it will be an anode, and negative output will appear (Figure S14).

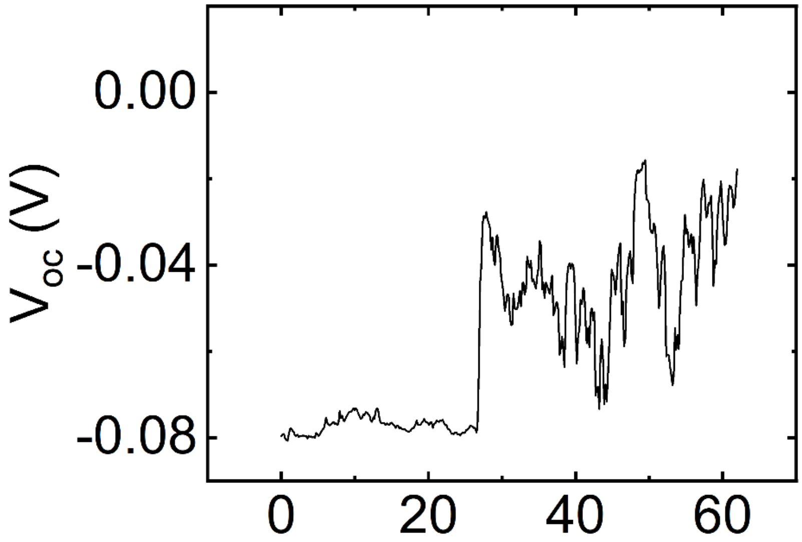

Figure S14. EC signals of Zn in pair of finished Al

SEM and EDX of textile in Al-Al pair under dry condition

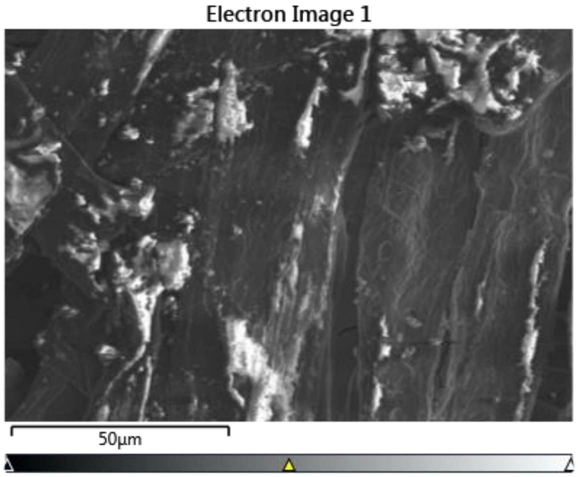

Figure S15. SEM of textile interface 1 in Al-Al (dry) pair

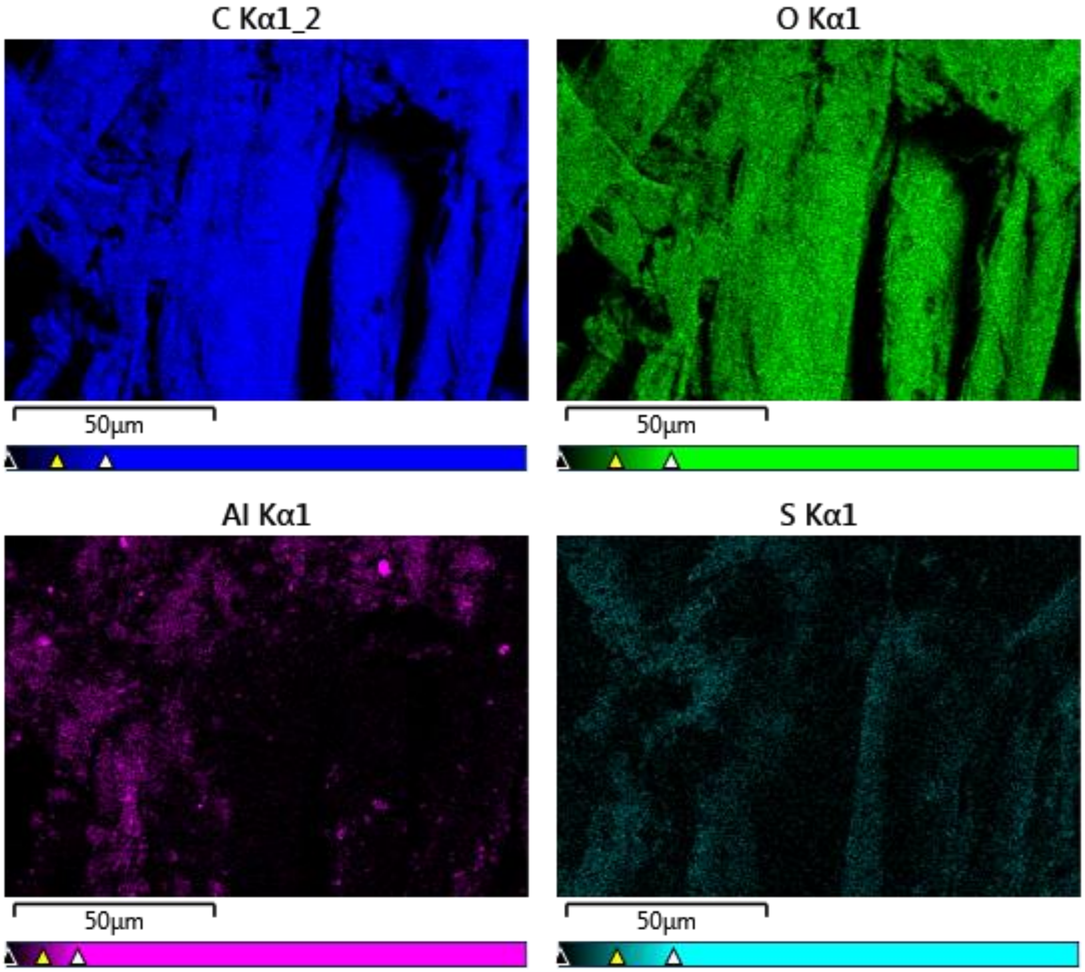

Figure S16. EDX mapping of textile interface 1 in Al-Al (dry) pair

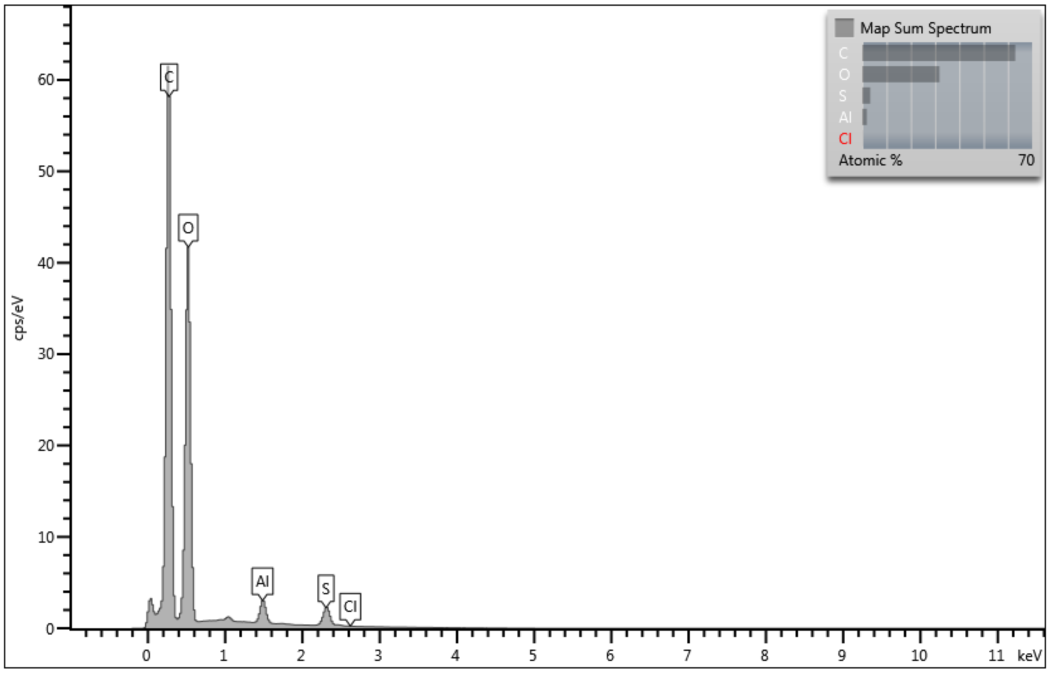

Figure S17. EDX spectrum of textile interface 1 in Al-Al (dry) pair

Al is found in interface 1 in Al-Al(dry) pair, which comes from the friction between the working electrode and textile. The materials consumption of the electrode here is identified as mechanical loss (Figure S15-S17). Less Al is found in interface 2 due to the less relative displacement between the collecting electrode and textile (Figure S18-S20).

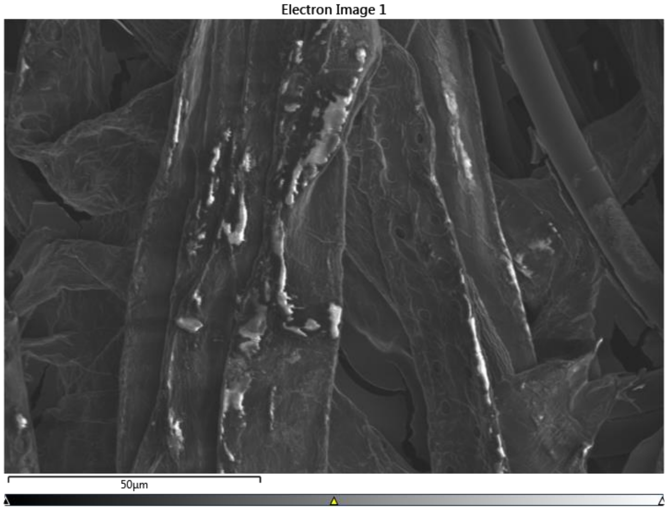

Figure S18. SEM of textile interface 2 in Al-Al (dry) pair

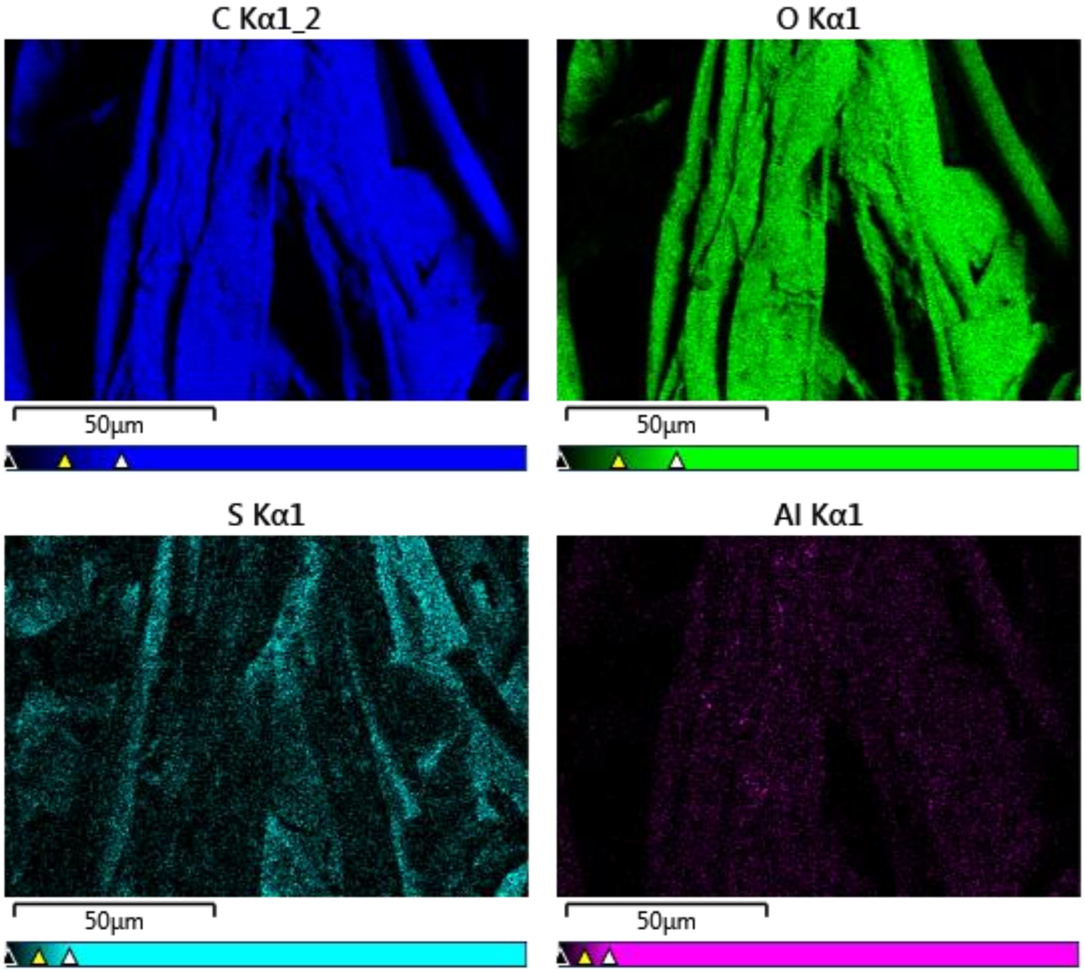

Figure S19. EDX mapping of textile interface 2 in Al-Al (dry) pair

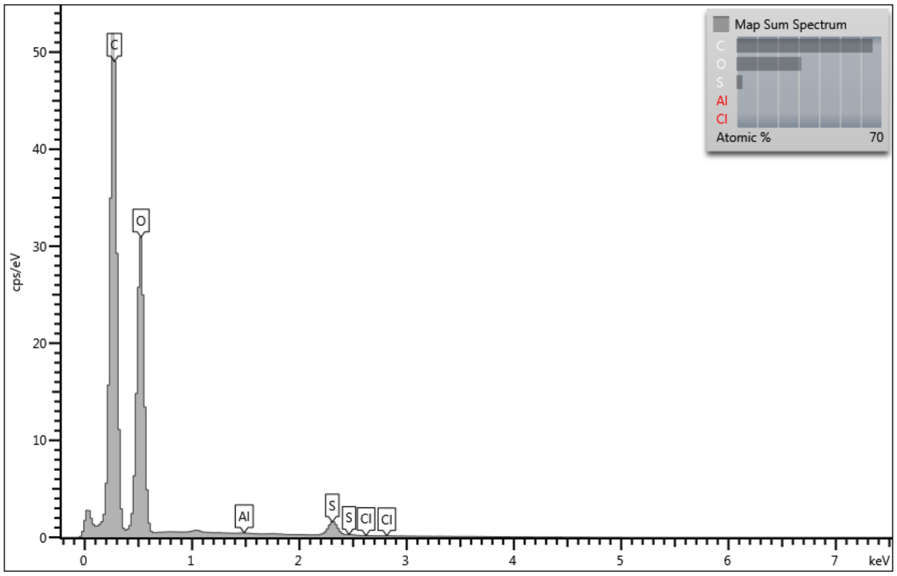

Figure S20. EDX spectrum of textile interface 2 in Al-Al (dry) pair

SEM and EDX of textile in Al-Al pair under wet condition

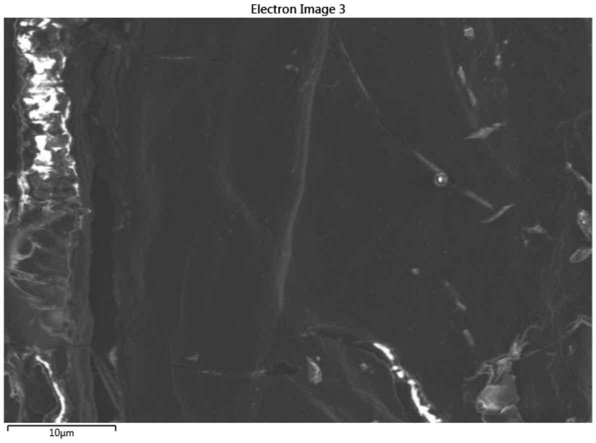

Figure S21. SEM of textile interface 1 in Al-Al (wet) pair

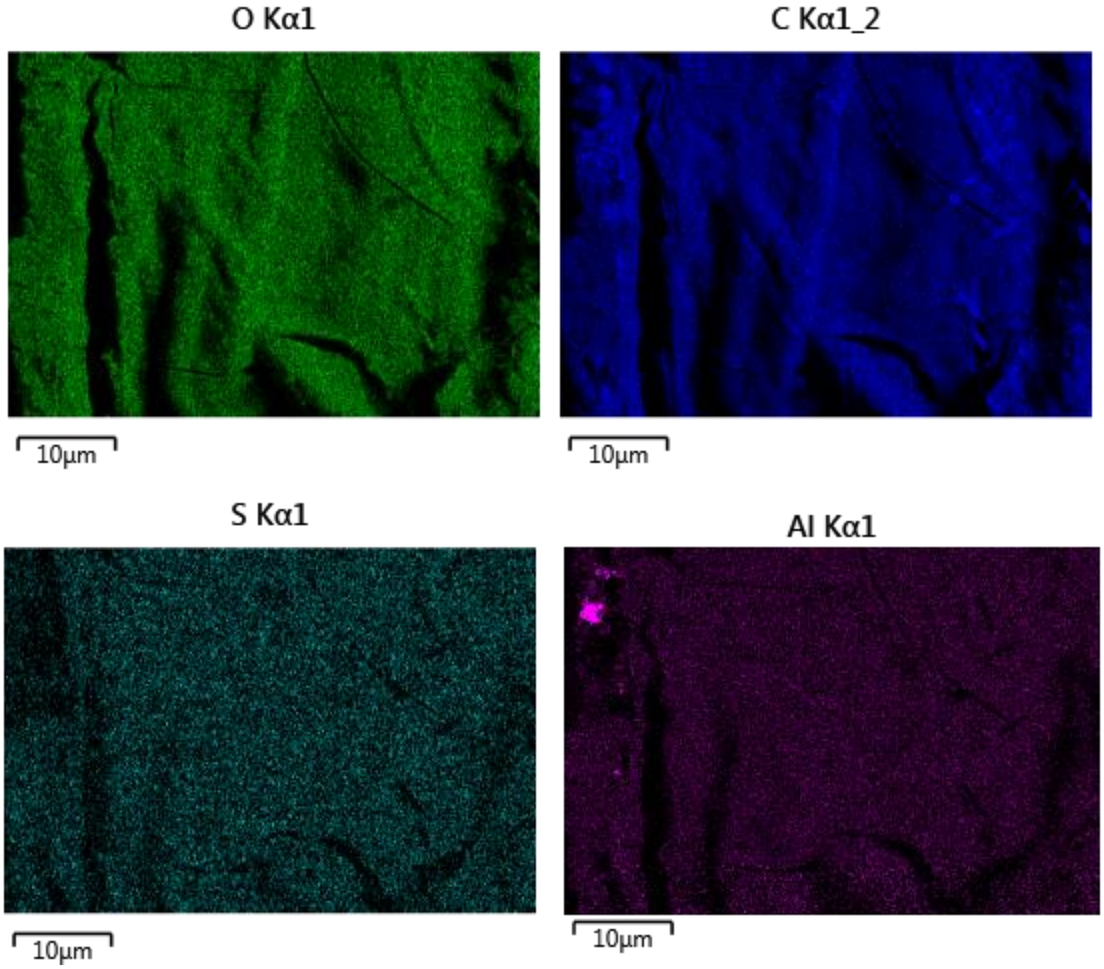

Figure S22. EDX mapping of textile interface 1 in Al-Al (wet) pair

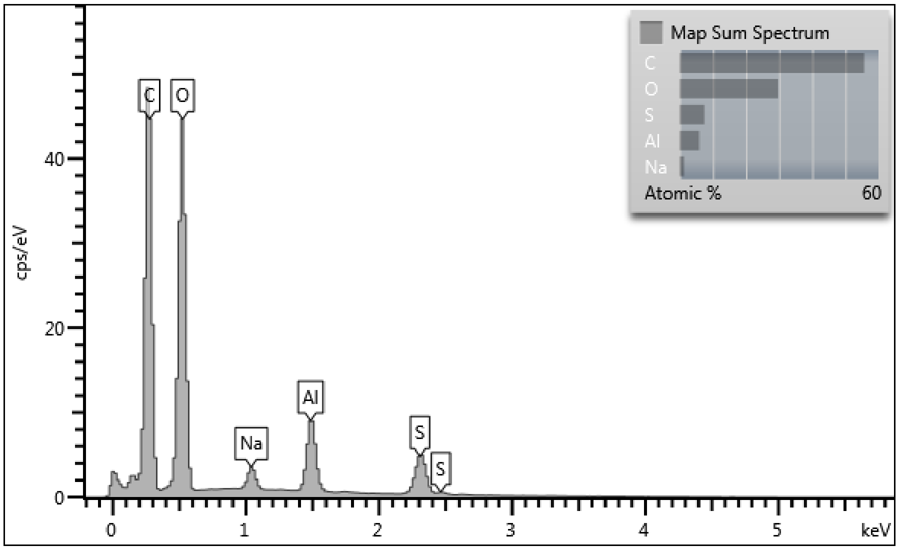

Figure S23. EDX spectrum of textile interface 1 in Al-Al (wet) pair

Al is found in both interfaces 1 & 2 in the Al-Al (wet) pair, which comes from the EC reaction and the generation of Al^{3+} between the working electrode and textile. The Al^{3+} can move in water within the textile, thus leading to its existence in interface 2. Compared to Al-Al (dry) pair, the Al consumption is significantly greater, revealing a dominant consumption of EC reactions.

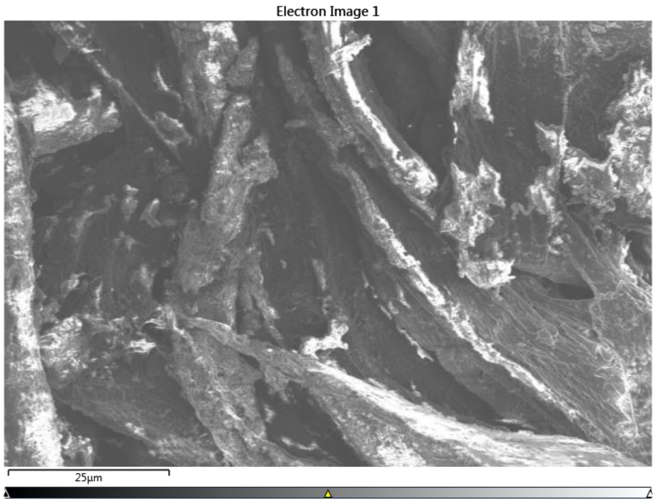

Figure S24. SEM of textile interface 2 in Al-Al (wet) pair

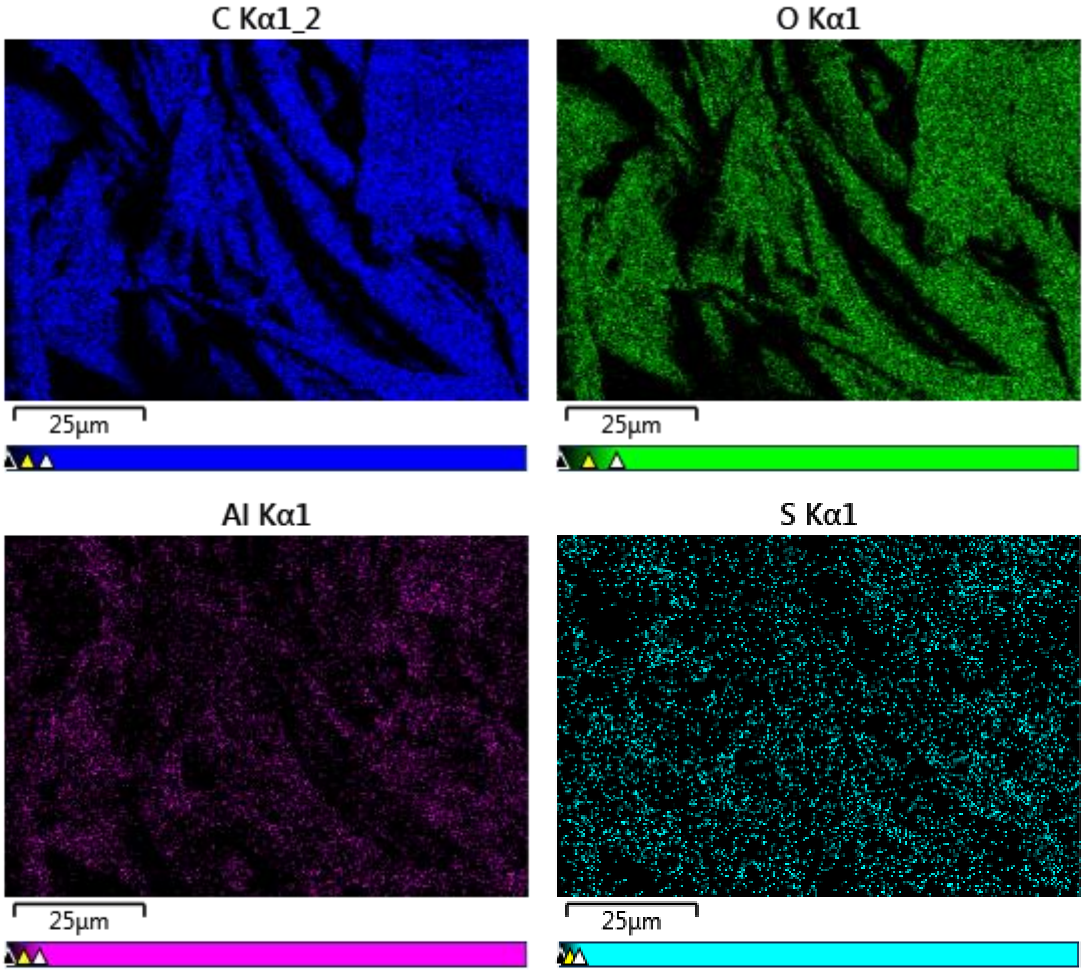

Figure S25. EDX mapping of textile interface 2 in Al-Al (wet) pair

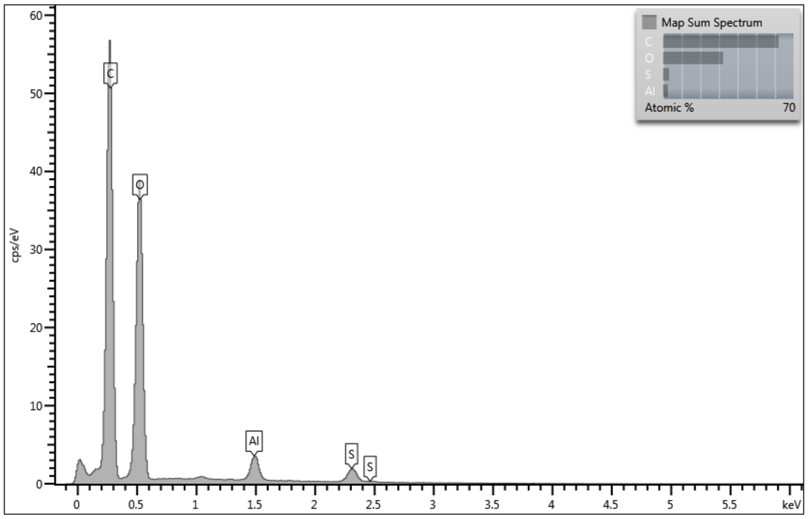

Figure S26. EDX spectrum of textile interface 2 in Al-Al (wet) pair

SEM and EDX of textile in Al-Ni pair under wet condition

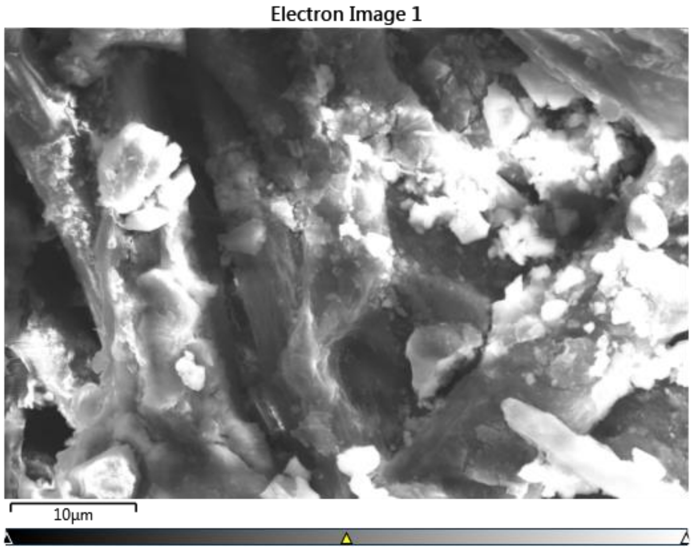

Figure S27. SEM of textile interface 1 in Al- Ni (wet) pair

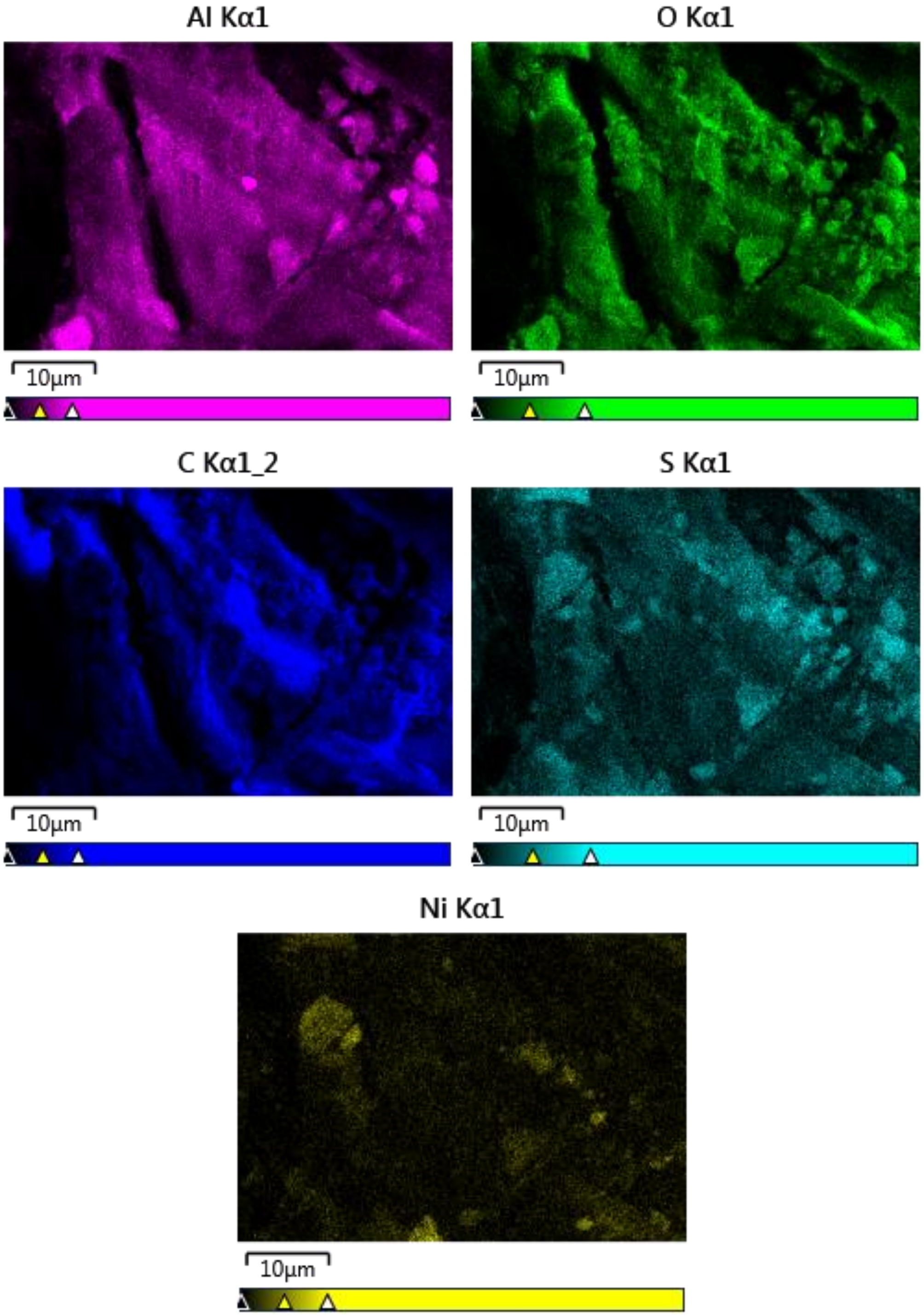

Figure S28. EDX mapping of textile interface 1 in Al-Ni (wet) pair

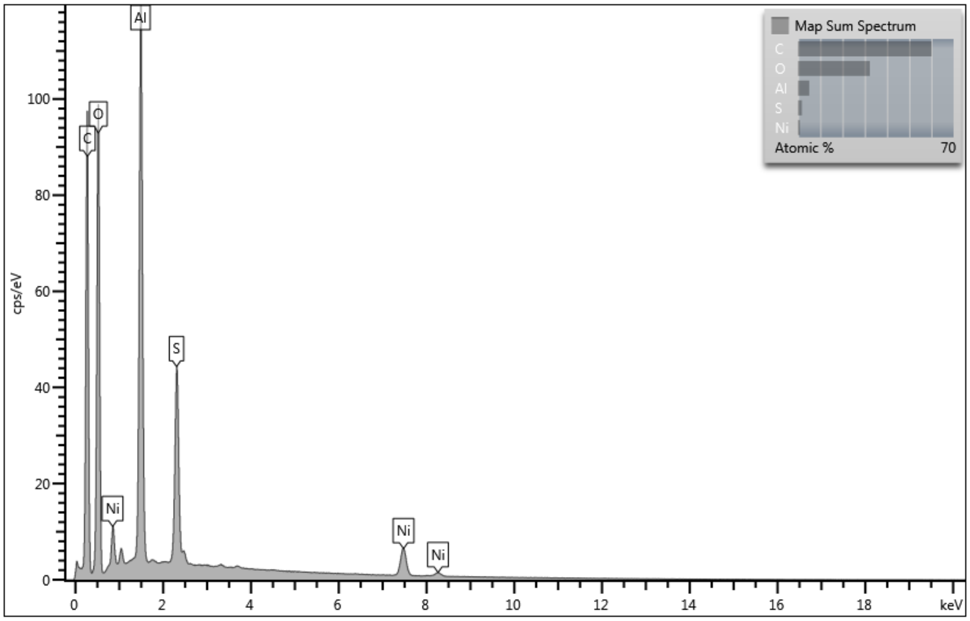

Figure S29. EDX spectrum of textile interface 1 in Al- Ni (wet) pair

Like the Al-Al (wet) pair, Al in the working electrode serves as the anode; thus, Ni is protected. Al is significantly found in both interfaces 1 & 2 in the Al-Ni (wet) pair, which comes from the EC reaction and the generation of Al^{3+} between the collecting electrode and textile. The Al^{3+} can move in water within the textile, thus leading to its existence in interface 2. Due to the larger differences between the two electrodes, the EC reactions will be more intensive. As a consequence, the Al is found to be consumed more than in Al-Al (wet) pair.

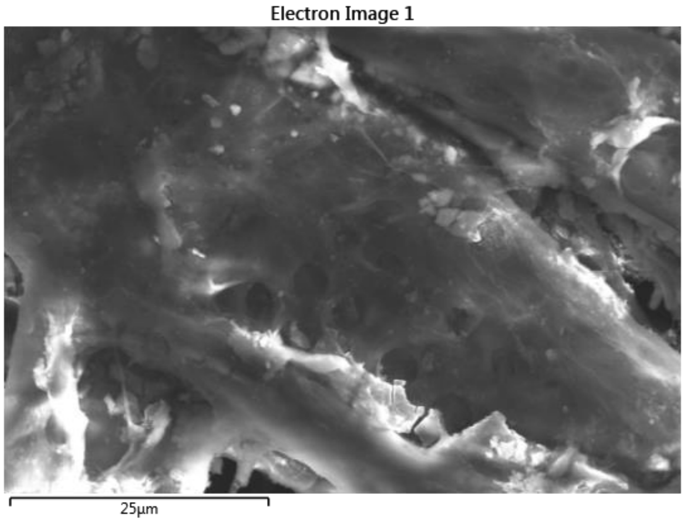

Figure S30. SEM of textile interface 2 in Al-Ni (wet) pair

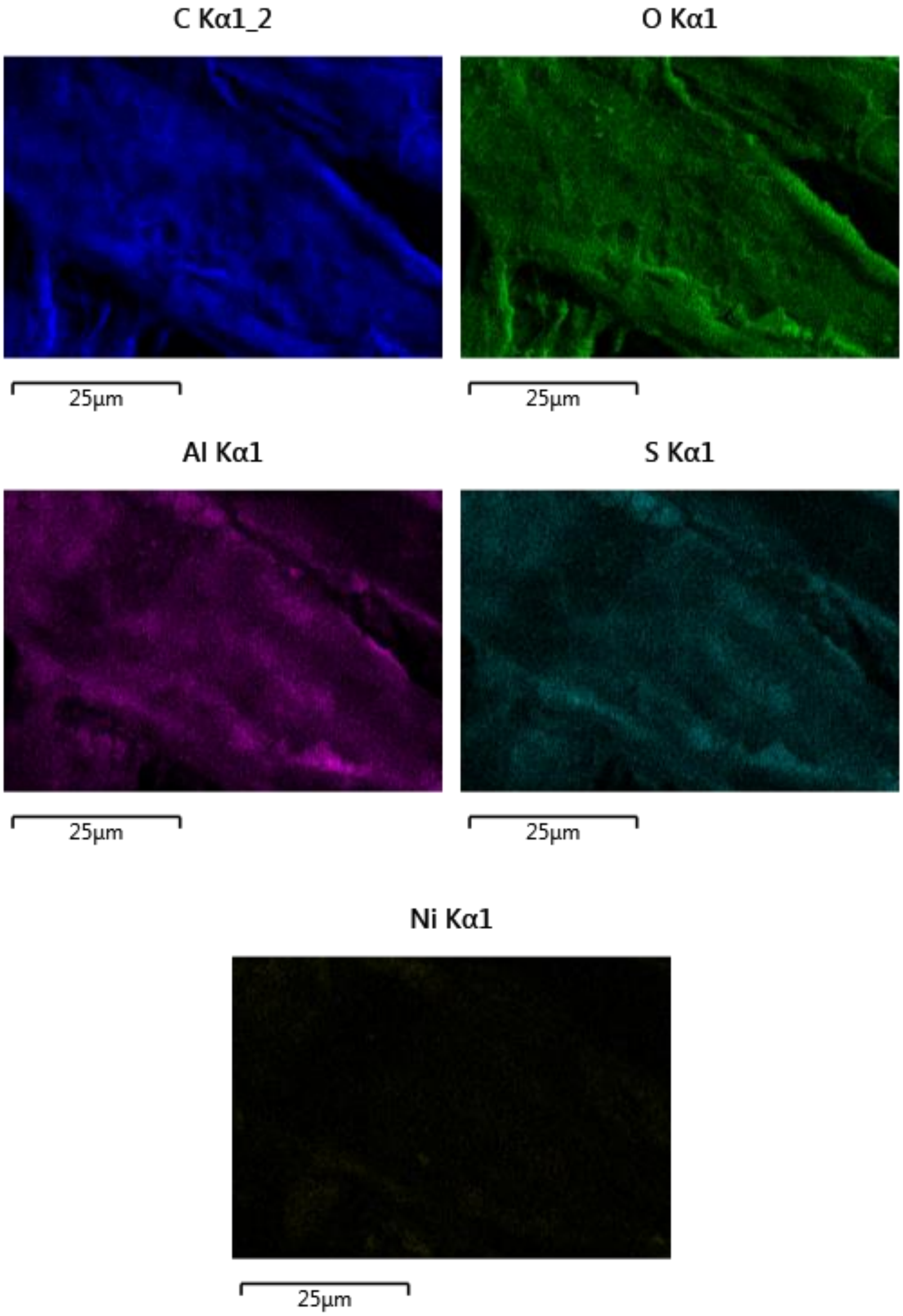

Figure S31. EDX mapping of textile interface 2 in Al-Ni (wet) pair

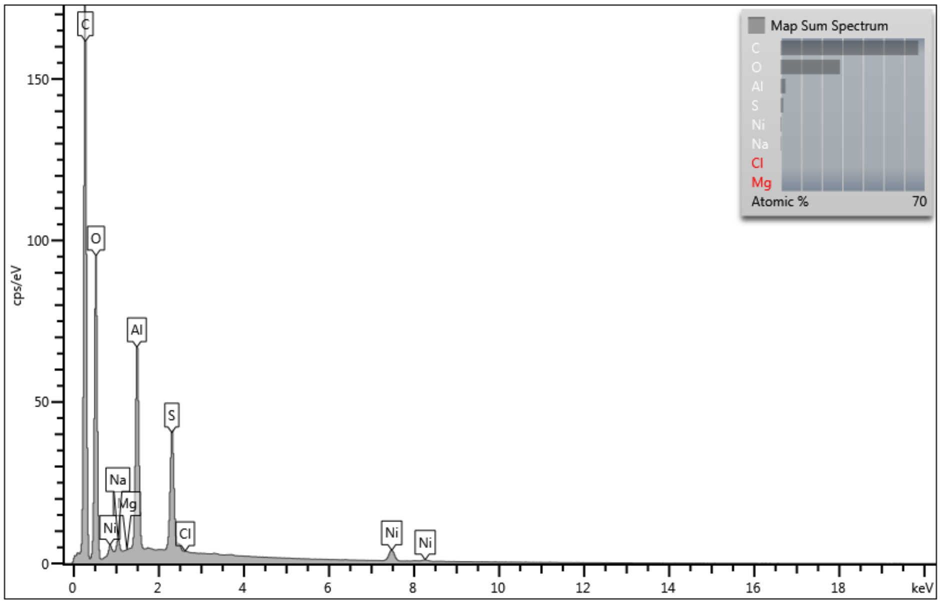

Figure S32. EDX spectrum of textile interface 2 in Al- Ni (wet) pair

<u>**SEM and EDX of textile in Al-Zn pair under wet condition**</u>

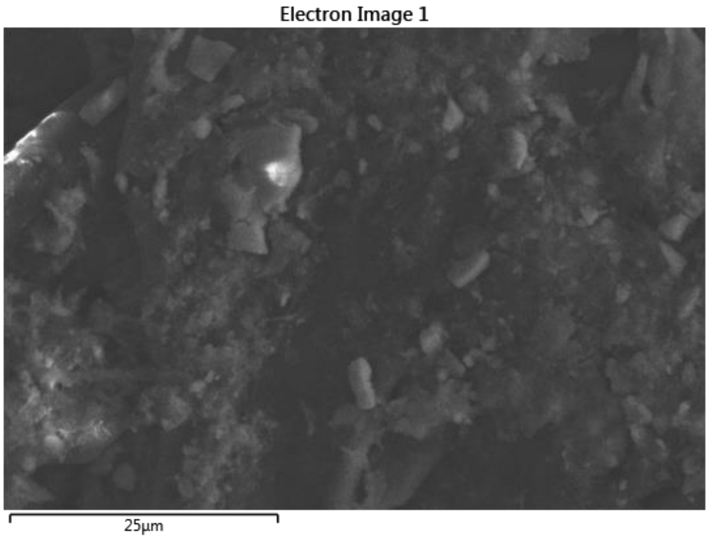

Figure S33. SEM of textile interface 1 in Al-Zn (wet) pair

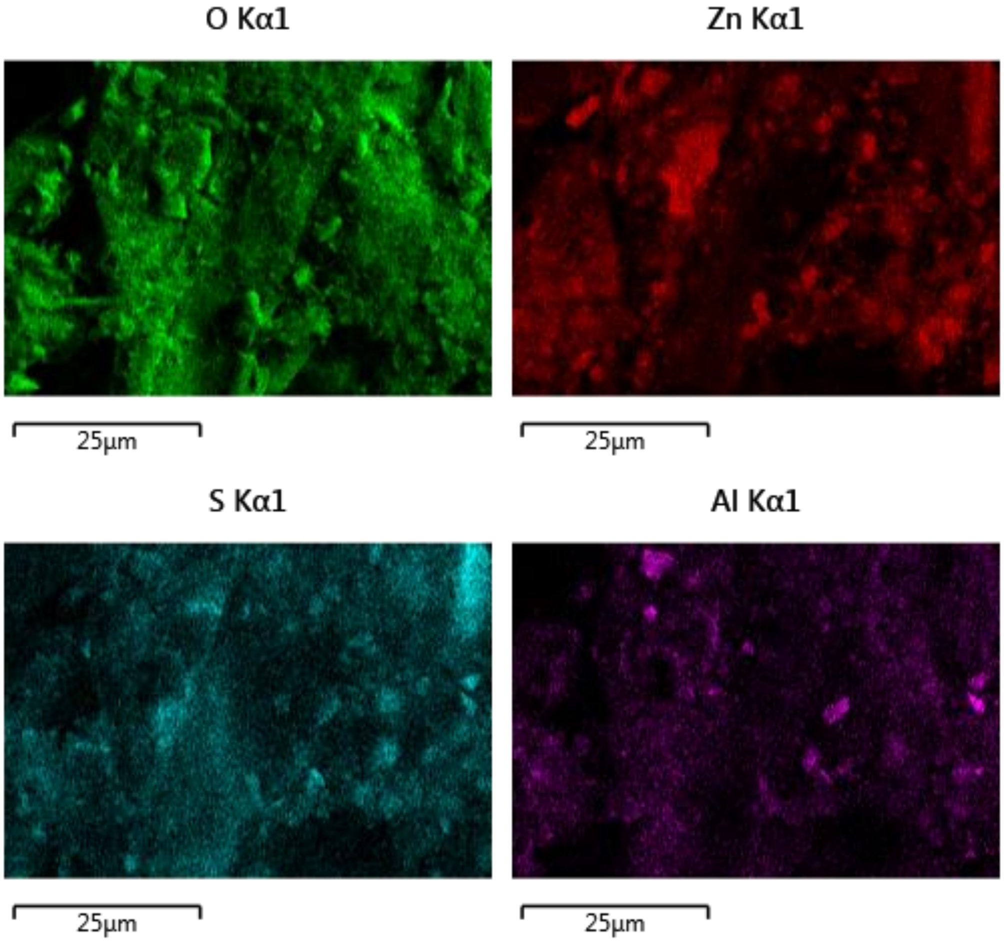

Figure S34. EDX mapping of textile interface 1 in Al- Zn (wet) pair

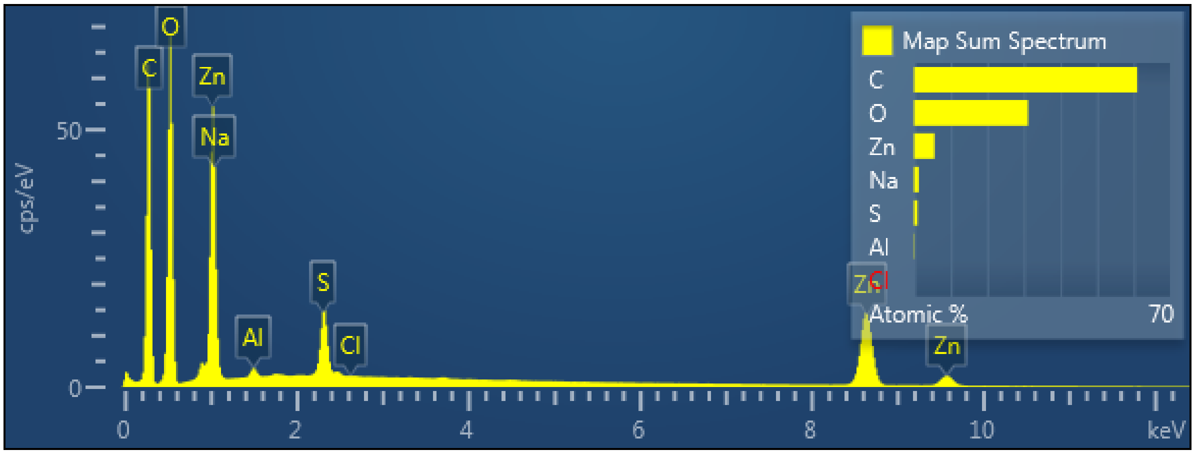

Figure S35. EDX spectrum of textile interface 1 in Al- Zn (wet) pair

Compared to the Al-Ni (wet) pair, Zn is significantly found in both interfaces 1 & 2 in the Al-Zn (wet) pair, which comes from the EC reaction and the generation of Zn^{2+} between collecting electrode and textile. The Zn^{2+} can move in water within the textile, thus leading to its existence in interface 1. Meanwhile, the Al will be protected by the Zn electrode while Zn works as an anode here, accounting that Al is found to be less than that in Al-Al (wet) pair. This phenomenon is in line with the previous direction of the Al-Zn pair.

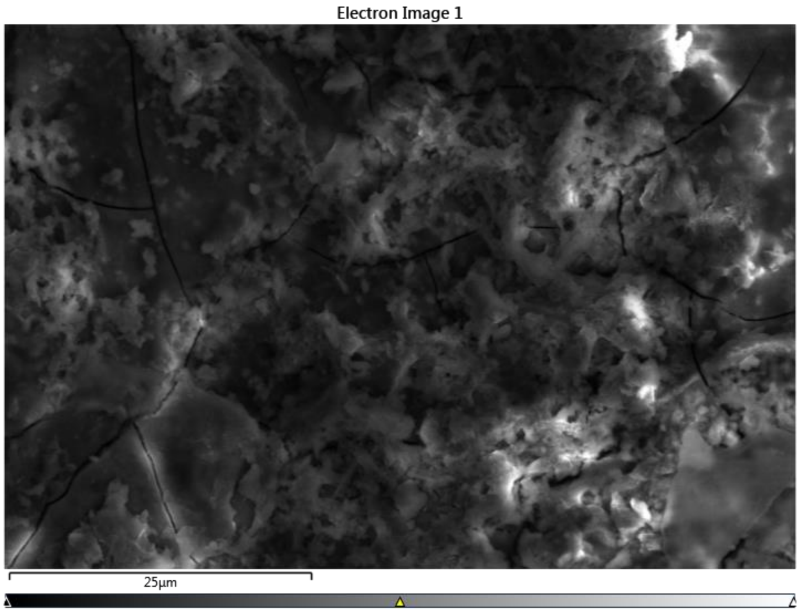

Figure S36. SEM of textile interface 2 in Al- Zn (wet) pair

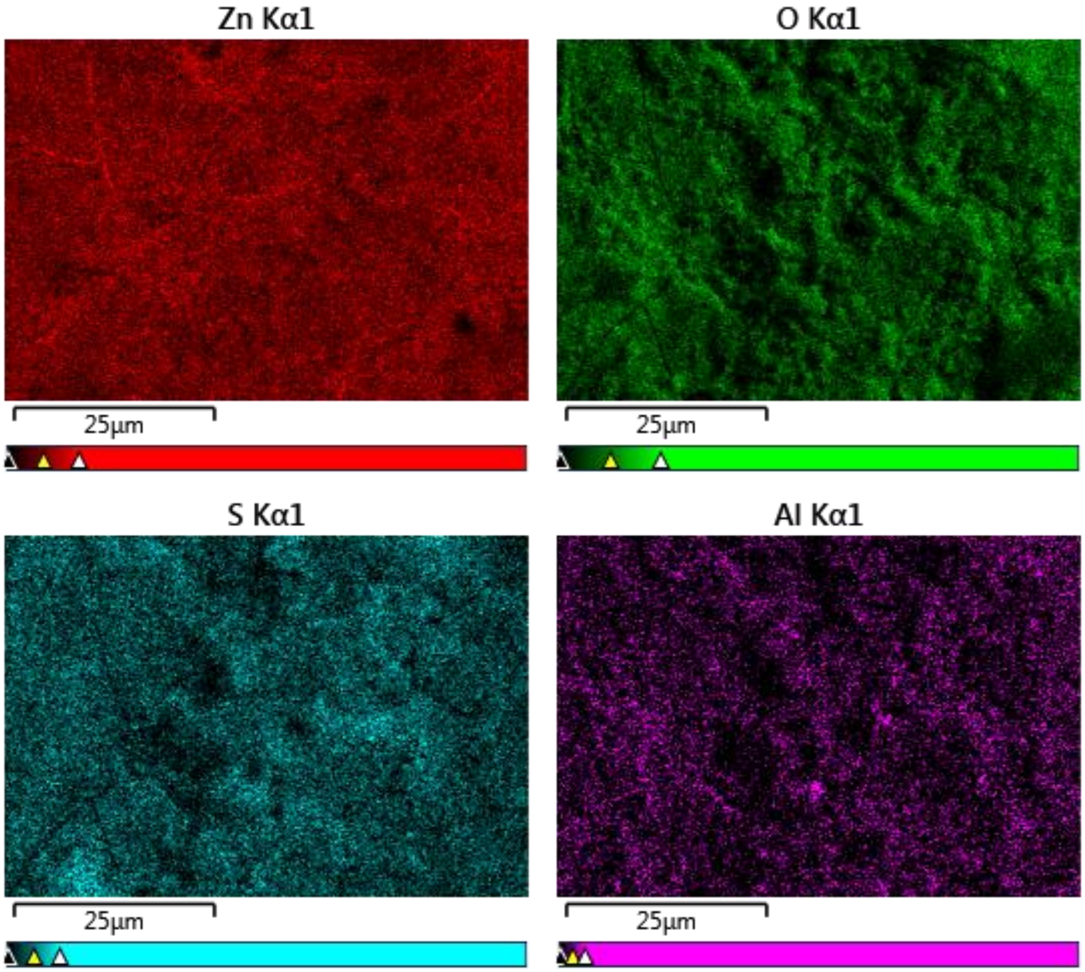

Figure S37. EDX mapping of textile interface 2 in Al- Zn (wet) pair

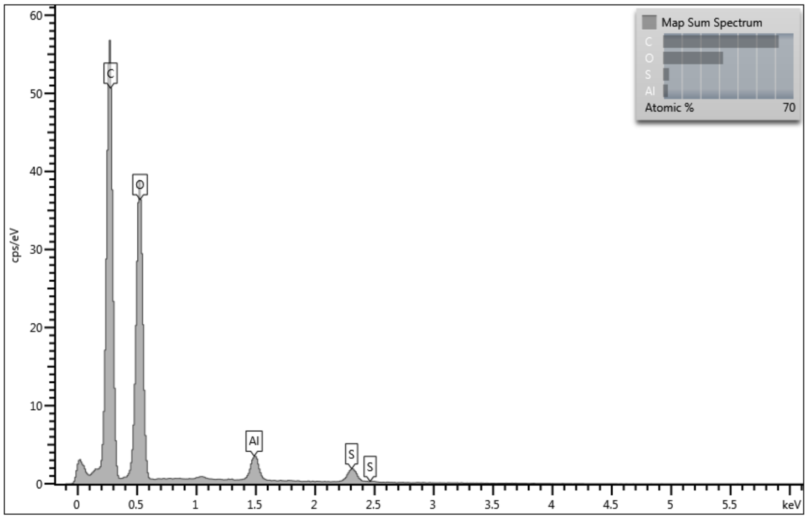

Figure S38. EDX spectrum of textile interface 2 in Al- Zn (wet) pair

Electrode metal selection

Experiments of the comparison of the influence of Al-Al, Zn-Zn, and Ni-Ni are conducted. It's shown in Figure S39 that the Al-Al pair has the largest output compared with the other two, specifically in the case that PEDOT; PSS works as the organic semiconductor.

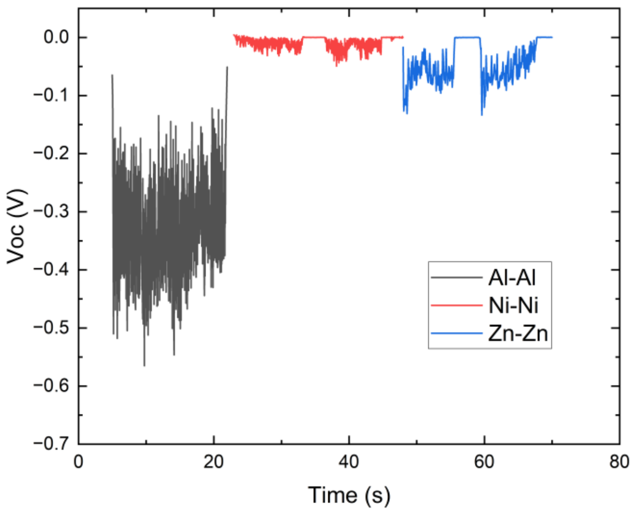

Figure S39. The output of DSD signals in different identical counter-electrode pairs

Washability test

To test the washing durability of the textile device, we stirred our samples in beakers at 600 rpm for 1h to imitate the laundry washing for each cycle. The samples are prewashed for 30 min before the washing cycle to remove the materials that are deposited on the surface of the textile. All the samples have been washed for 3h in total. After each cycle, the sample was air-forced dried in an oven at 100 °C and annealed on the hot plate at 100 °C for 15 min. After drying, the DC outputs are measured. Photos of samples after each cycle are shown in Figure S40. Partial PEDOT: PSS on the surface has been washed away after several hours, as the textile turns white in some areas. However, most PEDOT: PSS still adhered to the cotton threads even after 3h wash due to the bonding between PEDOT: PSS and textile. The DC outputs after each cycle are shown in Figure S41. After washing, the open-circuit voltage output decreases. Because some PEDOT: PSS at the interface has been washed, the carrier density decreases. Meanwhile, the effective contact area of PEDOT: PSS and metals reduce, leading to fewer carriers transfers between the interface and the lift of the Femi energy level in PEDOT: PSS. As the Femi energy level of PEDOT: PSS lifts, the mismatch between metals and PEDOT: PSS gets decreased, thus lower potential barriers are obtained, resulting in less open-circuit voltage. However, after 3h washing, the output still shows the level of 0.2 V as most PEDOT: PSS still bonds with the textile and is capable of forming Dynamic Schottky Diode. For further enhancement of washability, superhydrophobic treatment such as fluoroalkyl silane[2], fluorinated polymer sponge[3,4], or surface encapsulation such as silicone[5] and rubber[6] sealing can be applied.

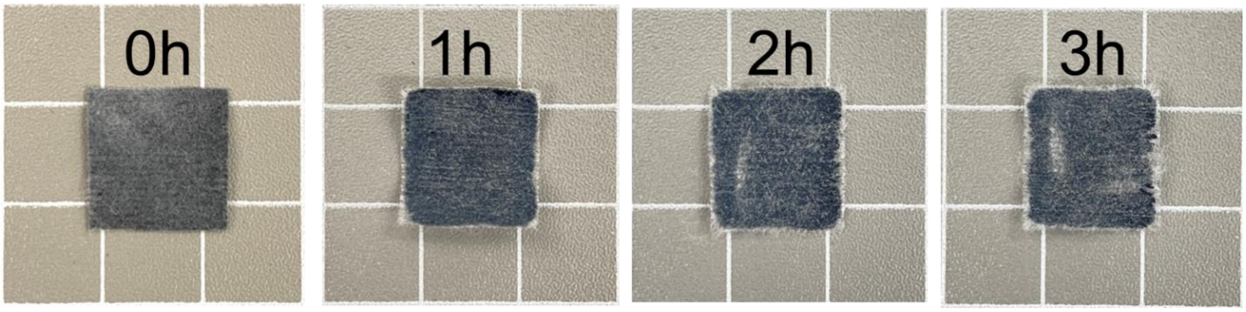

Figure S40. Photos of a nonwoven textile device after washing

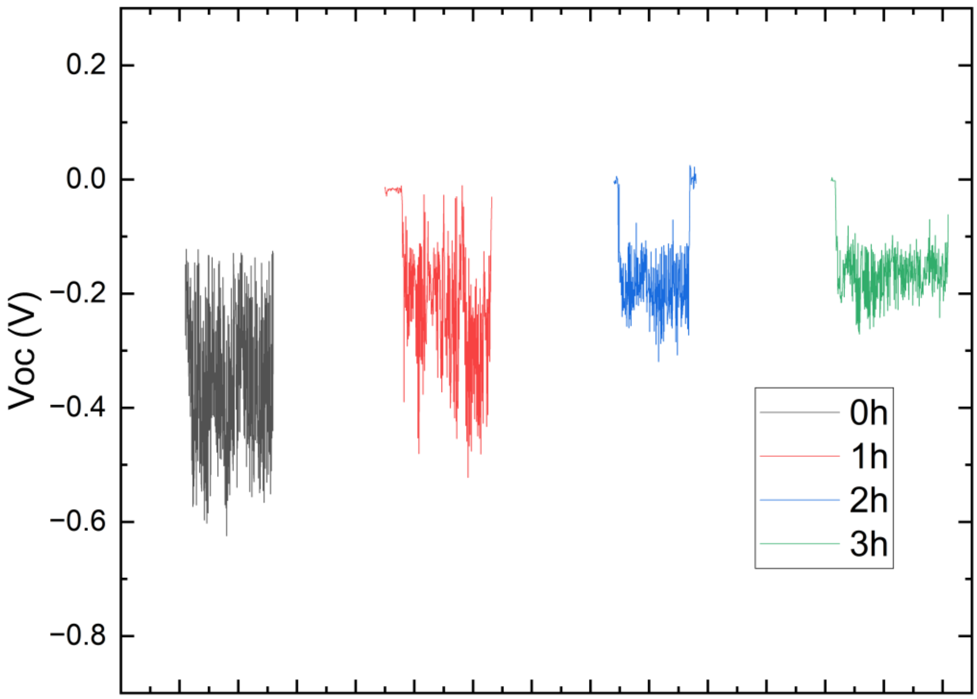

Figure S41. Output of DSD signals after washing

References

[1] D. Nilsson, N. Robinson, M. Berggren, R. Forchheimer, *Adv. Mater.* **2005**, *17*, 353.
[2] H. Zhou, H. Wang, H. Niu, A. Gestos, X. Wang, T. Lin, *Adv. Mater.* **2012**, *24*, 2409.
[3] X. Yan, X. Xiao, C. Au, S. Mathur, L. Huang, Y. Wang, Z. Zhang, Z. Zhu, M. J. Kipper, J. Tang, J. Chen, *J. Mater. Chem. A* **2021**, *9*, 21659.
[4] Z. Peng, J. Song, Y. Gao, J. Liu, C. Lee, G. Chen, Z. Wang, J. Chen, M. K. H. Leung, *Nano Energy* **2021**, *85*, 106021.
[5] E. Davoodi, H. Montazerian, R. Haghniaz, A. Rashidi, S. Ahadian, A. Sheikhi, J. Chen, A. Khademhosseini, A. S. Milani, M. Hoorfar, E. Toyserkani, *ACS Nano* **2020**, *14*, 1520.
[6] Y. Zou, A. Libanori, J. Xu, A. Nashalian, J. Chen, *Research* **2020**, *2020*, 1.
